# Microbiota transplantation and multi-omics profiling integration unveil the mechanism of *Alistipes communis*-driven abdominal fat deposition in chickens

**DOI:** 10.1186/s40104-026-01439-2

**Published:** 2026-05-20

**Authors:** Yang Jing, Shuai Liu, Li Leng, Jiarui He, Tianqi Wang, Yunnan Guan, Zhiyong Su, Wenpeng Zhang, Yumao Li, Peng Luan, Bohan Cheng, Ning Wang, Hui Li

**Affiliations:** 1 College of Animal Science and Technology, Northeast Agricultural University, Harbin, 150030 People’s Republic of China; 2 Key Laboratory of Chicken Genetics and Breeding, Ministry of Agriculture and Rural Affairs, Harbin, 150030 People’s Republic of China; 3 Key Laboratory of Animal Genetics, Breeding and Reproduction, Education Department of Heilongjiang Province, Harbin, 150030 People’s Republic of China

**Keywords:** Abdominal fat deposition, *Alistipes communis*, Cecal microbiota transplantation, Chicken, Multi-omics, Tryptophan and histidine metabolism

## Abstract

**Background:**

Emerging evidence highlights strong correlations between the cecal microbiome and abdominal fat deposition (AFD) in chickens. However, the specific microbial species driving this process remain unclear. This study aims to identify the key microbe and elucidate its underlying mechanism in regulating chicken AFD.

**Results:**

First, cecal microbiota transplantation confirmed a causal relationship between the cecal microbiota and AFD. Subsequently, metagenomic and metatranscriptomic integrations identified *Alistipes communis* as a key microbe implicated in AFD. Finally, in vivo gavage integrated with multi-omics revealed that *A. communis* enhances AFD by disrupting host tryptophan and histidine metabolism. This was evidenced by the elevated concentrations of amino acid metabolism-related metabolites, including L-phosphoarginine and spermine in the cecum.

**Conclusions:**

This study provides direct evidence that the cecal microbiome serves as a key driver in chicken AFD and identifies *A. communis* as a critical AFD regulator, offering valuable insights into the gut microbiome’s role in host obesity.

**Supplementary Information:**

The online version contains supplementary material available at 10.1186/s40104-026-01439-2.

## Background

The holobiont concept defines that animals are superorganisms composed of their own cells and symbiotic microbiota [1]. Together, the microbiota—collectively known as the microbiome—and the host genome shape phenogenesis, pathogenesis, and adaptive evolution in holobionts [2–4]. Among these symbionts, the gut microbiome plays a pivotal role in regulating host obesity [5, 6]. Disruptions or imbalances in gut microbial communities can significantly alter lipid metabolism, leading to abnormal fat deposition. Studies in humans [7], mice [8], pigs [9] and chickens [10] have shown substantial differences in the diversity, abundance, and functional composition of the gut microbiome between obese and lean individuals. The gut microbiome influences host lipid metabolism through several possible mechanisms, such as polysaccharide fermentation, glutamine synthesis, and chronic inflammation [9, 11]. However, the complex regulatory pathways involved remain poorly understood. Unraveling these mechanisms will improve our understanding of gut microbiome functionality and support the development of targeted strategies for the prevention and treatment of obesity and related metabolic diseases.

The cecum, a key site for water and electrolyte absorption, also serves as a hub for microbial fermentation and the retention of digestive products [12, 13]. The resident symbiotic cecal microbiota has been increasingly implicated in host lipid metabolism and obesity. For instance, early studies have demonstrated that transplanting cecal microbiota from conventional to germ-free mice suppressed *Fiaf* expression in the intestinal epithelium, increased lipoprotein lipase (LPL) activity, and promoted fat accumulation [14]. Subsequent research has highlighted the cecal microbiome’s regulatory role in obesity. In high-fat diet-induced obese mice, cecal *Akkermansia* exacerbates obesity by synthesizing glycerophosphodiesterase, which augments choline production and valyl-tRNA synthetase synthesis, thereby accelerating L-valine degradation [15]. The abundances of *Ileibacterium valens* and *Ruminococcus gnavus* were also found to be increased in obese mice, along with an elevation in bile and cholic acid levels in their cecum [16]. Metagenome-wide association reveled that interactions between cecal microbiota and host genetics regulate fat deposition in pigs, with *Paraprevotella* abundance being significantly correlated with body fat percentage [17]. In chickens, *Methanobrevibacter*, *Spirochaeta* and *Parabacteroides* have been identified as AFD-associated cecal microbial taxa [10, 18]. Furthermore, *Bacteroides* and *Parabacteroides* exhibited a significant negative correlation with abdominal fat percentage (AFP) in chicken, while several genera in Firmicutes exhibited a significant positive correlation [19]. Our previous study also demonstrated that the cecal microbiome had close relationships with broiler AFD [20]. Despite identifying numerous microbial genera, functional genes, and metabolites associated with host lipid metabolism, most current studies remain at the level of correlational analyses using omics data, and the exact mechanisms through which specific cecal microbes and their metabolites regulate lipid metabolism in animals remain unclear.

Excessive AFD in modern broilers poses major challenges, including reduced meat quality, compromised reproductive performance, and economic losses to the poultry industry [21]. Given the pivotal role of the cecal microbiota in shaping this trait [18, 19, 22], clarifying the underlying regulatory mechanisms offers a promising avenue for controlling fat deposition in broilers. Based on the previously observed differences in the cecal microbiome, we hypothesize that specific cecal microbe may influence abdominal fat deposition by modulating host physiological metabolism through their metabolites in chicken. To test this hypothesis, we designed three parts of experiments: cecal microbiota transplantation (CMT), integrated metagenomic (MG) and metatranscriptomic (MT) sequencing, and single-strain gavage. The findings from this study are expected to provide important insights into the role of gut microbiota in regulating abdominal fat deposition and to identify potential microbial biomarkers for early detection of excessive abdominal fat in chickens. These insights may also have broader implications for understanding obesity in mammals.

## Methods

### Experimental animals and experimental design

AA chickens and fat and lean broilers from the 26^th^ generations of the Northeast Agricultural University broiler lines divergently selected for abdominal fat content (NEAUHLF) were used in this study. The lines have been selectively bred since 1996, with AFP and plasma very low-density lipoprotein (VLDL) concentration as the primary selection criteria, as described previously [23]. The 26^th^ generation of the NEAUHLF displays over a 13-fold difference in AFP between the lean and fat lines, providing an ideal model for studying adiposity and obesity in chickens. All chickens used for cecal microbiota transplantation were reared in a same standardized poultry facility with ad libitum access to water and feed. To prevent microbiota cross-contamination via fecal droppings, the chickens were housed in separate, single-layer cages. The birds were fed a commercial soybean-based diet formulated to meet the National Research Council (NRC) nutritional requirements [24]. A starter diet containing 3,000 kcal metabolizable energy (ME)/kg and 210 g/kg crude protein (CP) was provided from hatch to 3 weeks of age, followed by a grower diet with 3,100 kcal ME/kg and 190 g/kg CP from 3 to 7 weeks of age [23]. Chickens subjected to strain-specific microbiota gavage were housed in negative-pressure sterile isolators with high-efficiency particulate arrestance (HEPA) filtration. They received autoclaved drinking water (121 °C/15 min) and γ-irradiated feed (25 kGy, Co^60^ source) to eliminate microbial contaminants. At 7 weeks of age, the experimental chickens were euthanized through cervical dislocation to facilitate sample collection for subsequent analyses.

### Cecal microbiota transplantation

Male chickens from the 26^th^ generation of the 7-week-old NEAUHLF lean- and fat-lines were used as microbiota donors. Thirty individuals were selected from extreme families of each line. Fresh cecal contents were collected immediately post-slaughter using sterile scoops, transferred into sterile 50-mL centrifuge tubes, and diluted in sterile PBS containing 10% glycerol at a 1:10 g/mL ratio [25]. The contents were homogenized, centrifuged at 279 × *g* for 10 min, and filtered through three layers of sterilized gauze and filter cloths (0.2 and 0.1 mm) to produce a uniform brown microbial suspension. The combined inoculum from all 30 donors of each line was mixed in a single beaker, mixed thoroughly, and then aliquoted into 5-mL frozen storage tubes for storage at −80 °C one week until use. All procedures were performed on ice inside an anaerobic chamber to preserve microbial viability.

The recipient chickens were commercial AA males, divided into four groups (*n* = 5 per group): Blank (without any treatments), Control (gavaged with PBS), LLA (gavaged with lean-line donor cecal microbiota suspension), and FLA (gavaged with fat-line donor cecal microbiota suspension). During the first week post-hatch, all chickens (except those in the Blank group) were administered 10 mL/kg/d of a mixed broad-spectrum antibiotic solution (containing neomycin sulfate, ampicillin, metronidazole, vancomycin, and amphotericin B at 0.5 g/L each) to minimize microbial interference from vertical transmission and the hatching environment [26]. Subsequently, the cecal contents from the respective donors were orally inoculated into their corresponding recipient groups. From weeks 2 to 4, each chicken received 1 mL/d of the cecal microbiota suspension (previously stored in a −80 °C freezer and thawed at 37 °C for 30 min). In weeks 5 and 6, inoculations were administered three times per week (on 29, 31, 33, 36, 38, and 40 d) to enhance microbial colonization. No treatments were administered during week 7. At the end of week 7, all chickens were sacrificed to obtain samples for abdominal fat phenotype measurements. The cecal contents from each group and the corresponding donor cecal contents were subjected to 16S rRNA sequencing to compare the microbial composition and assess microbiota colonization status in the recipient chickens. The cecal contents of all fat- and lean-line donors were also used for metagenomic and metatranscriptomic sequencing.

### 16S rRNA sequencing and analysis

Microbial DNA was extracted using the QIAamp^®^ Fast DNA Stool Mini Kit (Qiagen, Hilden, Germany) following the manufacturer's instructions. DNA concentration and purity were measured using a NanoDrop 2000 UV–Vis Spectrophotometer (Thermo Scientific, Wilmington, USA). DNA integrity was verified through 1% agarose gel electrophoresis. 16S rRNA gene sequencing was performed by Majorbio Bio-Pharm Technology Co., Ltd. (Shanghai, China) following established protocols [27]. Briefly, the V3–V4 hypervariable region of the bacterial 16S rRNA gene was amplified using 515F/806R primers. PCR products were purified and quantified using the AxyPrep DNA Gel Extraction Kit (Axygen Biosciences, CA, USA) and Quantus™ Fluorometer (Promega, WI, USA), respectively. Sequencing was performed on the Illumina MiSeq platform (Illumina, San Diego, USA). Raw fastq files were quality-filtered, and microbial diversity analysis was conducted using the Majorbio I-Sanger Cloud platform (https://cloud.majorbio.com).

### Metagenomic sequencing and analysis

As described above, total microbial DNA was extracted and sheared to form approximately 350-bp fragments by using a Covaris M220 ultrasonicator (Gene Company Limited, China). Next, a paired-end library was constructed using the NEXTFLEX Rapid DNA-Seq Kit (Bio Scientific, USA), followed by metagenomic sequencing on the Illumina NovaSeq platform. Raw sequencing reads were quality-filtered using fastp, removing reads shorter than 50 bp, with low quality scores (< 20), or containing ambiguous N bases. High-quality pair-end and single-end reads were retained. Host-derived sequences were removed by aligning reads to the chicken genome using BWA, and contaminated reads were discarded. Filtered reads were assembled using MEGAHIT, retaining contigs ≥ 300 bp. Open reading frames (ORFs) were predicted using MetaGene, and genes ≥ 100 bp were translated into amino acid sequences. Redundant genes were clustered using CD-HIT (90% identity, 90% coverage) to create a non-redundant gene catalog. Taxonomic annotation and abundance profiling at various taxonomic levels were conducted using DIAMOND software with BLASTP against the NCBI-NR database (http://ab.inf.unituebingen.de/software/diamond/). Functional annotation was performed using the eggnog, CAZy, and KEGG pathway databases to assess gene content and pathway enrichment.

### Metatranscriptome sequencing

A 0.25 g sample of frozen cecal contents was collected, and total RNA was extracted using the RNeasy PowerMicrobiome Kit (Qiagen, Hilden, Germany). RNA quality was assessed through denaturing gel electrophoresis and a nucleic acid concentration analyzer to ensure integrity and compliance with quality standards. Ribosomal RNA (rRNA) was removed using the RiboMinus Transcriptome Isolation Kit (Thermo Fisher, MA, USA) for bacteria. The enriched mRNA was used to construct a MT library using the NEBNext Ultra RNA Library Prep Kit for Illumina (NEB, MA, USA), including fragmentation, cDNA synthesis, library construction, and purification. Raw sequencing data were processed using FastQC and Trimmomatic for adapter removal, filtering of low-quality reads, and elimination of sequences shorter than 150 bp or containing > 10% N bases. Clean reads were aligned to the latest chicken reference genome (version: GalGal7b) using STAR to remove host RNA contamination. Residual rRNA fragments were filtered out using SortmeRNA. De novo assembly of metatranscriptomic reads was conducted using Trinity, and ORFs were predicted using MetaGeneMark. Redundant sequences were clustered using CD-HIT to produce the final non-redundant metatranscriptomic gene set. This gene set was aligned to the NCBI-NR database using DIAMOND, and species annotations were performed using MEGAN's lowest common ancestor algorithm. Clean reads were aligned to the reference gene set for taxonomic profiling at various levels (kingdom, phylum, class, order, family, genus, species). Along with MG data, microbial diversity, abundance, and community structure were assessed to identify key functionally relevant microbes. Differential gene expression analysis was conducted using DESeq2, with functional annotation and enrichment conducted by referring to KEGG, eggNOG, and CAZy databases.

### *A. communis* gavage and functional exploration in vivo

Considering core metabolic functions are often conserved within a bacterial species, despite strain-level variations [28]. A commercial *A. communis* strain 5CBH24 (DSM: 108979, German Collection of Microorganisms and Cell Cultures) was cultured on Medium 78 solid culture medium per the manufacturer's protocol under strict anaerobic conditions. Three randomly selected colonies were identified via 16S rRNA sequencing (Table S1), and the remaining colonies were resuspended in sterile PBS to prepare microbiota gavage suspensions.

Fertilized eggs from a commercial AA broiler breed were incubated, and newly hatched chicks were transferred into sterile isolators at a P2-level biosafety laboratory of the Harbin Veterinary Research Institute, Chinese Academy of Agricultural Sciences (ethical approval number: 240508-01-GR). Sixty chicks were randomly assigned to four groups: BL (Blank, without antibiotics pretreatments), CNAb (PBS control gavage with antibiotic pretreatment for 1 week post-hatch), AGAb (*A. communis* gavage with antibiotic pretreatment for 1 week post-hatch), and AG (oral gavage with *A. communis* without antibiotic pretreatment). Each experimental group had two isolators. The CNAb and AGAb groups received broad-spectrum antibiotics during the first week post-hatching to suppress endogenous microbiota, whereas the BL and AG groups received no treatment. From weeks 2 to 6, oral gavage was performed by using a two-phase administration strategy: (1) continuous administration (7–28 d), the AG and AGAb groups received 1 mL/kg/d of *A. communis* suspension; and (2) intermittent administration (29–42 d), the AG and AGAb groups received 1 mL/kg of *A. communis* suspensions three times per week (on 29, 31, 33, 36, 38, and 40 d). Synchronously, the CNAb group received an equal volume of sterile PBS, whereas the BL group received no treatment. At 7 weeks of age, the chickens were fasted for 12 h, weighed, and sacrificed. Abdominal fat tissues were dissected, weighed for abdominal fat weight (AFW), and prepared for histological analysis. The AFP was calculated as AFW/BW. Six recipient chickens were selected from each group, and blood was collected from the wing vein for serum metabolomics analysis. Cecal contents and mucosal tissues of same individuals were collected from the same individuals for 16S rRNA, MG, MT, and metabolomic sequencing. All sequencing followed the aforementioned methods.

### Histological measurements and analysis

Adipose tissues and cecum from the same six chickens per group in the *A. communis* gavage experiment were immediately fixed in 10% neutral-buffered formalin for 48 h at room temperature. Paraffin sections were prepared, stained with hematoxylin and eosin (H&E) [29], and scanned using a Digital Pathology Scanner 400 (Ningbo Konfoong Bioinformation Tech Co., Ltd., China) to generate whole-slide images. Image analysis was performed using KFBIO Digital Slide viewer software. For each individual’s adipose tissue, approximately 100 adipocytes from three fields were randomly selected for measuring the area of each adipocyte. Assuming that adipocytes are spherical, the average diameter ($\overline{d }$) and standard deviation ($s$) of adipocytes were calculated based on these areas. Then, the volume of each adipocyte was calculated according to the cell volume formula [30]:

$$\overline{V }=\frac{1}{6}\pi \left(3{s}^{2}\overline{d }+{\overline{d} }^{3}\right)$$

Using a human fat density of 0.915 g/mL, the total number ($N$) of adipocytes in the abdominal adipose tissue of each individual was calculated as follows [31]:

$$N=\frac{W}{0.915\overline{V} }$$

where $W$ represents abdominal fat weight and $\overline{V }$ is the average volume of each adipocyte. For scanned images of each individual's cecum, five complete, neatly arranged and longest villi and deepest crypts were selected to measure villus length, villus middle width, villus area, the number of goblet cells per villus, crypt depth, and villus length/crypt depth [32]. Additionally, mucosal and muscle layer thicknesses were measured at three random points per slide, with triplicate measurements averaged for comparison.

### Cecal content and serum metabolomics sequencing and analysis

A 100-μL aliquot of cecal content or serum was transferred into a 1.5-mL centrifuge tube, followed by the addition of 400 μL extraction solvent (acetonitrile:methanol = 1:1). The mixture was vortexed for 30 s, ultrasonically extracted at 5 °C (40 kHz) for 30 min, cooled at −20 °C for 30 min, and centrifuged at 13,000 × *g* for 15 min at 4 °C. The supernatant was evaporated to dryness under nitrogen. The residue was reconstituted in 120 μL of acetonitrile:water (1:1), ultrasonically extracted for 5 min (5 °C, 40 kHz), and centrifuged at 13,000 × *g* for 5 min at 4 °C. The supernatant was collected into vials with inserts for LC–MS/MS analysis. LC–MS/MS was performed using a Thermo UHPLC-Exploris480 system equipped with an ACQUITY HSS T3 column (100 mm ×2.1 mm i.d., 1.8 μm; Waters, USA) at Majorbio Bio-Pharm Technology Co., Ltd. (Shanghai, China).

Quality control (QC) samples were prepared from pooled extracts of equal volumes of metabolites from all samples and analyzed alongside test samples to assess reproducibility. LC–MS raw data were processed using Progenesis QI software for baseline filtering, peak detection, integration, retention time correction, and peak alignment, resulting in a data matrix containing mass-to-charge ratios and peak intensities. Variables with non-zero values in at least 80% of the samples were retained. Missing values were imputed with the minimum value. Peak intensities were normalized, yielding a normalized data matrix. Variables with relative standard deviations (RSD) > 30% in the QC samples were excluded, and data were log10-transformed to produce the final matrix for analysis. Metabolites were annotated by matching MS and MSMS spectra data against the HMDB and Metlin databases. Differential metabolites were identified using VIP scores from the OPLS-DA model and *P* values from Student’s *t*-test. Pathway enrichment analysis was conducted using the scipy.stats module in Python, with biological pathways identified using Fisher’s exact test.

### Mucosa transcriptomic sequencing and analysis

To ensure consistency, cecal mucosal samples were uniformly collected from the middle segment of the cecum in all experimental chickens. Total RNA was extracted from cecal mucosal tissue using the TRIzol method. The concentration and purity of the extracted RNA were assessed using a NanoDrop 2000 spectrophotometer. RNA integrity was evaluated through agarose gel electrophoresis, and the RNA integrity number was determined using an Agilent 5300 Bioanalyzer. The library construction requirements included total RNA ≥ 1 μg, concentration ≥ 50 ng/μL, and OD260/280 ratio between 1.8 and 2.2. The mRNA was enriched from total RNA by using magnetic beads with oligo (dT) primers targeting the poly (A) tails. Subsequently, cDNA synthesis was performed using the SuperScript double-stranded cDNA synthesis kit (Invitrogen, CA, USA). The cDNA libraries were constructed following Illumina’s protocol, including end-repair, phosphorylation, A-tailing, and adapter ligation. Fragment size selection (~300 bp) was performed through 2% Low-Range Ultra Agarose gel electrophoresis, followed by 15 cycles of PCR amplification using Phusion DNA polymerase (NEB). After quantification using a Qubit 4.0 fluorometer, the libraries were sequenced using the NovaSeq X Plus sequencer platform (2 × 150 bp, paired-end) at Majorbio Bio-Pharm Technology Co., Ltd. (Shanghai, China). After sequencing, raw sequencing reads were quality-filtered to obtain high-quality clean reads for subsequent analysis. These reads were then aligned to the chicken GRCg7b genome (https://www.ncbi.nlm.nih.gov/datasets/genome/GCF_016699485.2/) for transcript assembly and expression quantification. Quality metrics included sequencing saturation, gene body coverage, read distribution across different genomic regions, and read distribution across different chromosomes. Finally, gene expression analysis, differential gene expression analysis, and differential gene functional enrichment analysis were conducted using RSEM (http://deweylab.github.io/RSEM/), DESeq2 (http://bioconductor.org/packages/stats/bioc/DESeq2/), and Python scipy package (https://scipy.org/install/), respectively. The differential expression genes with |log_2_FC| ≥ 1 and FDR < 0.05 (DESeq2) were considered significantly differentially expressed.

### Real-time quantitative PCR

Total RNA was extracted from the liver and abdominal fat tissues of six recipients from each of the AGAb and CNAb groups by using TRIzol reagent (Invitrogen, Carlsbad, CA, USA) following the manufacturer’s protocol. Genomic DNA was removed, and reverse transcription was performed using the PrimeScript™ RT reagent kit with gDNA Eraser (Perfect Real Time; Takara Bio Inc., Kusatsu, Japan). Primers specifically targeting lipid metabolism-related marker genes (Table S2) were designed, and only those with amplification efficiencies between 90% and 110% were used [33]. Quantitative PCR was performed on an ABI QuantStudio™ 6 Flex Real-Time PCR System (Applied Biosystems, Foster City, CA, USA) by using the FastStart SYBR Green Master (Roche, Mannheim, BW, Germany). The reaction conditions were as follows: initial denaturation at 95 °C for 10 min, 40 cycles of 95 °C at 15 s, and 60 °C for 1 min. The *TBP* gene was selected as the internal reference gene in qPCR experiments. Ct values of all genes were analyzed using the QuantStudio™ Real-Time PCR Software v1.3 (Applied Biosystems). Relative gene expression between the two groups was calculated using the 2^−ΔΔCt^ method.

### Statistical analysis

All data are presented as mean ± standard error of the mean (SEM). Comparisons of phenotypic and gene expression data were performed using Student’s *t*-test or one-way ANOVA in GraphPad Prism 9.0. Principal coordinate analysis (PCoA) based on Bray–Curtis dissimilarity was used to evaluate group differences. The vegan package in R (v2.5.4) was used for ANOSIM and PERMANOVA to assess variation between groups. Adjusted *P* values < 0.05 were considered statistically significant. All bioinformatics analyses were conducted using the cloud-based platform at https://cloud.majorbio.com/page/tools.html.

## Results

### Cecal microbiota transplantation and its effect on AFD in chickens

**Fig. 1:**
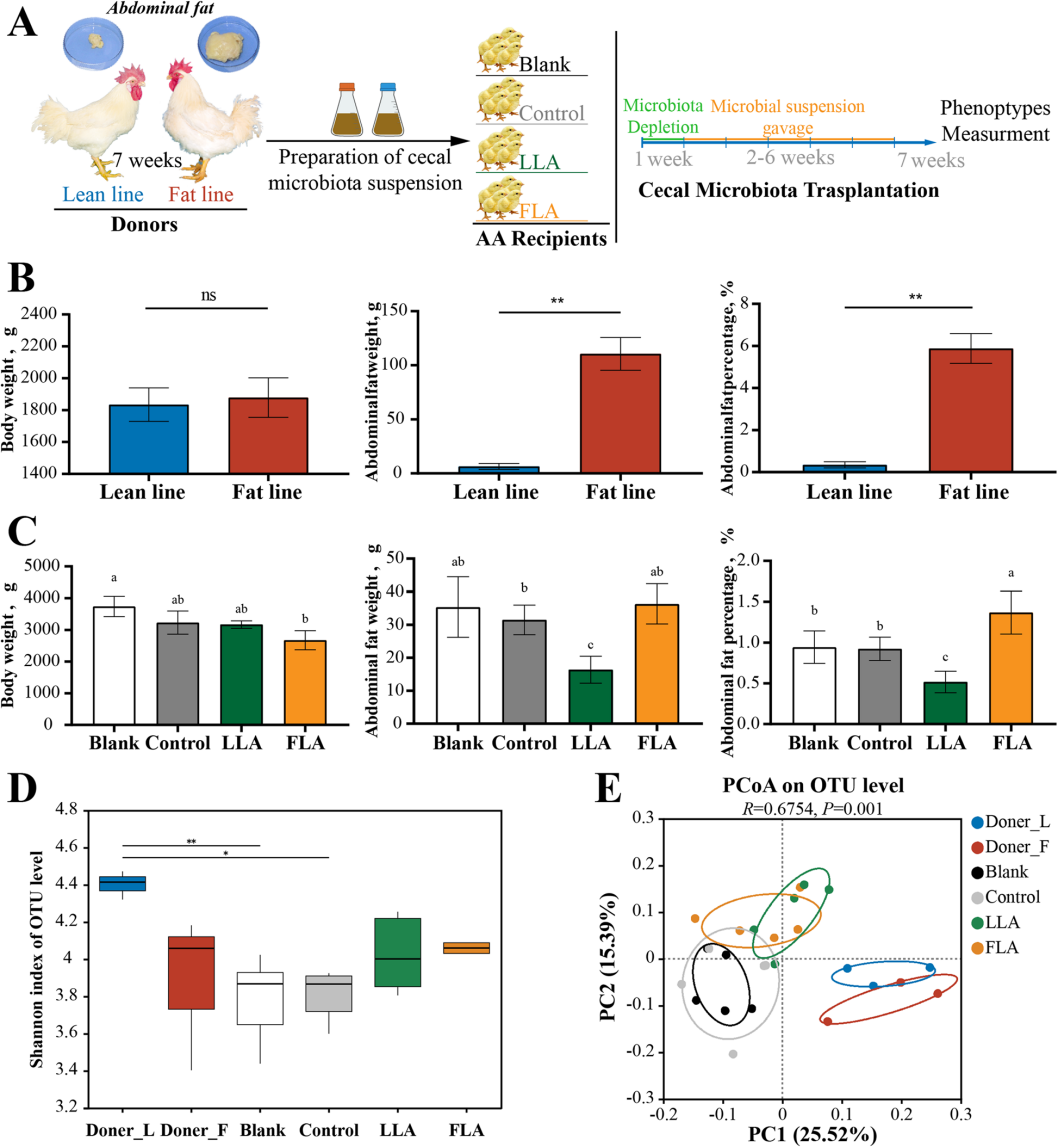
Impacts of cecal microbiota transplantation on recipient chicken phenotypes and cecal microbiome. **A** Schematic diagram of the cecal microbiota transplantation experiment. **B** and **C** Phenotypic comparisons of donor (*n* = 30) and recipient chickens (*n* = 5) for body weight (left), abdominal fat weight (middle), and abdominal fat percentage (right) at 7 weeks of age. **D** and **E** The α diversity and OTUs composition comparisons between donor and recipient chicken groups. Different letters indicate significant differences (*P* < 0.05), whereas same letters indicate no significant difference (*P* > 0.05). ns indicates no significance; * indicates *P* < 0.05, ** indicates *P* < 0.01. LLA, gavage group with lean-line donor cecal microbiota suspension; FLA, gavage group with fat-line donor cecal microbiota suspension

Cecal contents from thirty 7-week-old male chickens of both fat- and lean-lines were used as donor samples. Phenotypic analysis revealed no significant differences in body weight between the two donor lines (*P* > 0.05); however, both AFW and AFP were significantly higher in the fat-line donors (*P* < 0.01; Fig. 1B). Among AA recipients, only the FLA group showed a marked decrease in BW (*P* < 0.05), whereas the Control and LLA groups showed only a slight reduction relative to the Blank group (*P* > 0.05; Fig. 1C). However, direct comparisons among the three treated groups (Control, LLA, and FLA) revealed no significant differences in BW (*P* > 0.05; Fig. 1C). Taken together, the observed decrease in BW appears to be led by the gavage procedure itself, not by differences in the transplanted microbiota source. Regarding AFD traits, the FLA group exhibited a significant increase in AFP compared with the Control group, whereas the LLA group showed a significant reduction (*P* < 0.05; Fig. 1C). No significant differences in AFW or AFP were observed between the Control and Blank groups (*P* > 0.05; Fig. 1C), confirming that the gavage procedure alone did not influence AFD. 16S rRNA sequencing verified successful transplantation, as the microbial compositions of recipient groups showed a significant separation by their respective donors (Fig. 1D and E; Fig. S1; Tables S3 and S4). Collectively, these findings demonstrate that CMT from fat- and lean-line donors to AA recipients induced corresponding high- and low-AFP phenotypes, providing strong evidence for a causal relationship between the cecal microbiota and AFD in chickens.

### Integrative analysis to identify key microbial species associated with AFD

Based on 882.76 Gb of clean data obtained from MG sequencing of two lines’ donors, we constructed a non-redundant MG reference gene set comprising 3,359,811 unique genes and subsequently annotated 4,200 genera and 21,164 species (Table S5). Alpha diversity analysis showed significantly lower ACE and Chao indices in the fat-line chickens compared with the lean-line chickens (*P* < 0.05), along with distinct species composition between the two lines’ donors (*P* < 0.05; Fig. S2). MT sequencing yielded 1,012.14 Gb of clean data and 667,603 unique reference genes, allowing for the identification of 1,286 genera and 4,458 species (Table S6; Fig. S3). It also showed distinct MT taxonomic profiles between the two lines’ donors (Fig. S3E and F).

**Fig. 2:**
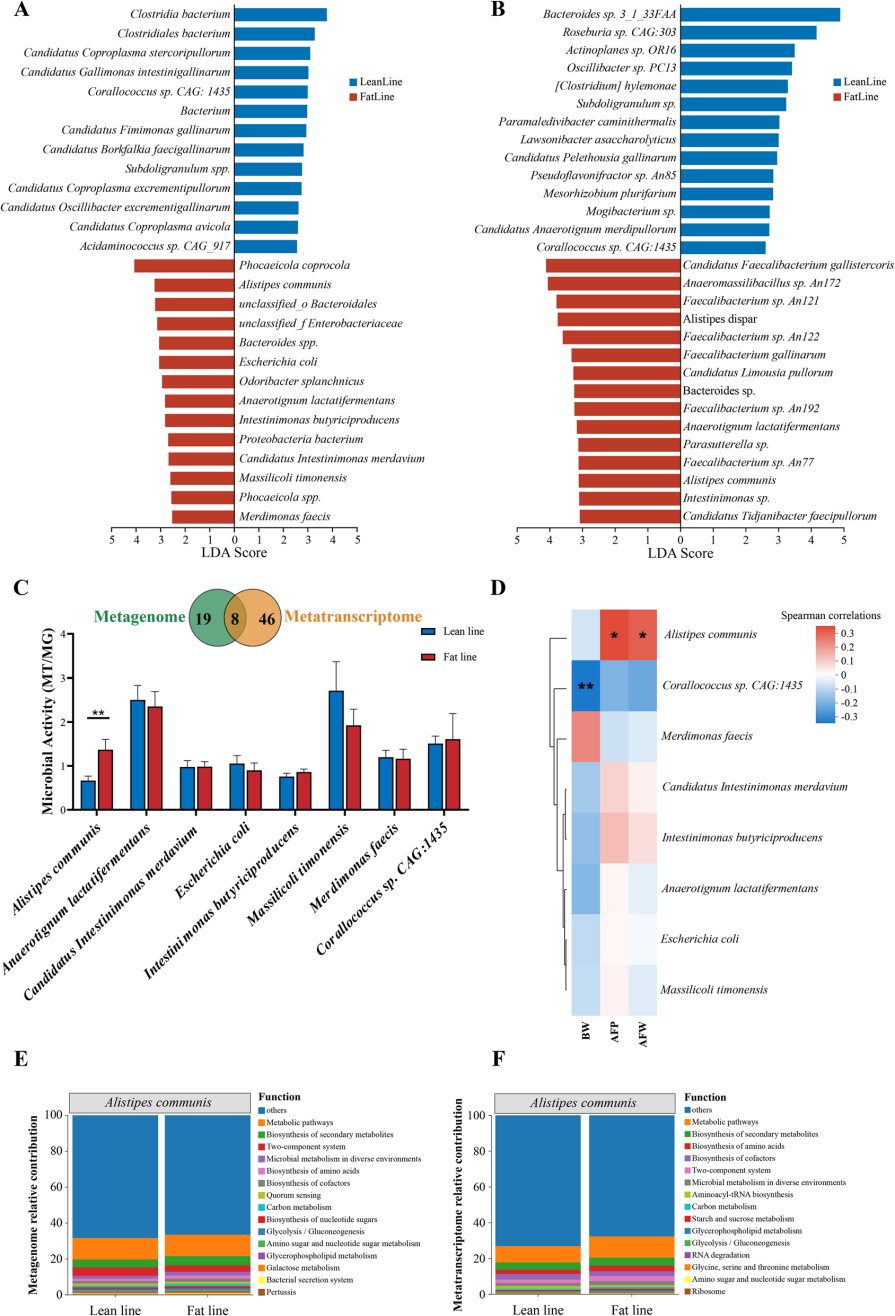
Integrated analysis of differentially abundant species from metagenomic and metatranscriptomic data. **A** Metagenomic (MG) differential species profiling (*n* = 30). **B** Metatranscriptomic (MT) differential species profiling (*n* = 30). **C** The upper Venn showing overlap of differentially abundant species between MG and MT datasets. The activity of these differentially abundant species was assessed by the ratio of MT to MG abundance. **D** Correlation heatmap showing associations between microbial activity and phenotypic traits; red color represents a positive correlation, whereas blue color represents a negative correlation. **E** and **F** Functional contribution plots of *Alistipes communis* based on MG and MT analyses, respectively. Differential species were identified using the LEfSe method (LDA > 2.5, *P* < 0.05). If fewer than 15 species were significant, all were displayed. The Mann–Whitney U test was used for significance testing of microbial activity; * indicates *P* < 0.05, ** indicates *P* < 0.01

Differential abundance analysis revealed 27 and 54 significantly varying species in the MG and MT datasets, respectively (LDA > 2.5, *P* < 0.05; Fig. 2A and B; Table S7). The intersection of the two datasets highlighted that eight species overlapped between the two datasets, of which seven were significantly enriched in the fat-line chickens compared with the lean-line chickens (LDA > 2.5, *P* < 0.05; Fig. 2A–C). Among them, *A. communis* was the only species with significantly higher relative abundance and transcriptional activity in fat-line chickens than in lean-line chickens (Fig. 2A–C). Furthermore, the *A. communis* activity exhibited a significant positive correlation with AFP and AFW (*P* < 0.05; Fig. 2D). Functional taxon-specific contribution analysis using MG and MT data showed that *A. communis* was predominantly involved in amino acid metabolism, cofactor biosynthesis, and the metabolism of carbon, amino/nucleotide sugars, and galactose (Fig. 2E and F). Overall, integrated MG and MT profiling accurately identified *A. communis* as a key active microbial species that plays a vital role in regulating AFD in chickens.

### Functional mechanism elucidation of *A. communis* in chicken AFD

**Fig. 3:**
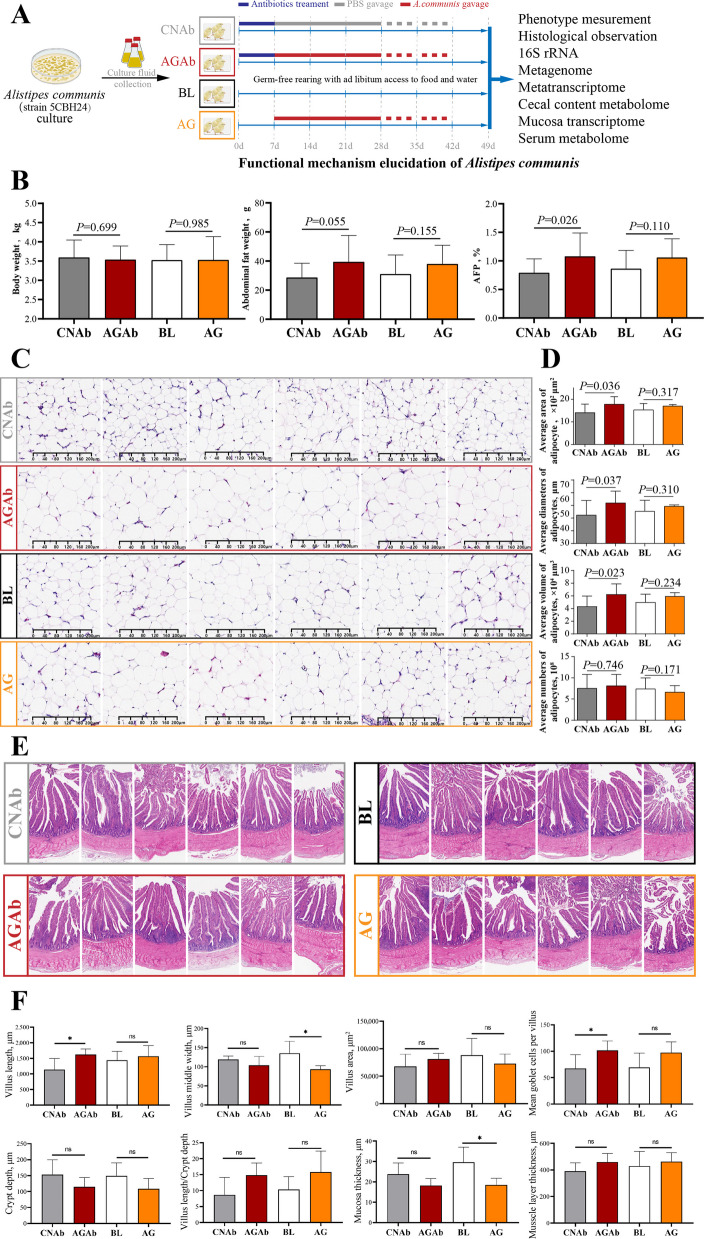
*A. communis* colonization and its effect on recipient phenotypes. **A** Schematic diagram of the *A. communis* gavage experiment. **B** The comparison of body weight (left), abdominal fat weight (middle), and abdominal fat percentage (right) among the four receipt groups; column with different letters indicate significant difference between the two groups (*n* = 15). **C** The H&E-stained section of abdominal fat tissue from the four groups (*n* = 6). **D** The comparison of adiocyte area (top), diameter (upper-middle), volume (lower-middle), and numbers (bottom) between the gavage and non-gavage groups. **E** The H&E-stained section of cecum from the four groups (*n* = 6). **F** The comparison of villus length, villus middle width, villus area, goblet cell number (the first line from left to fight), crypt depth, villus length/crypt depth, mucosal thickness, and muscle layer thickness (the second line from left to right) between the gavage and non-gavage groups. CNAb, PBS control gavage with antibiotic pretreatment for 1 week post-hatch; AGAb, *A. communis* gavage with antibiotic pretreatment for 1 week post-hatch; BL, blank; AG, *A. communis* gavage without antibiotic pretreatment. ns indicates no significance; * indicates *P* < 0.05

Single-strain gavage experiments were conducted, as presented in Fig. 3A. At 1 week of age, 16S rRNA sequencing of three random cecal samples from distinct groups confirmed that antibiotic treatment effectively depleted the cecal microbiota, establishing uniform baselines before *A. communis* gavage (Fig. S4). After gavage from 2 until 6 weeks of age, chickens were euthanized at 7 weeks of age for phenotypic and multi-omics data collection.

#### Impact of *A. communis* gavage on chicken AFP phenotype

Phenotypic analysis revealed that the AGAb group had a significantly higher AFP than the CNAb group (*P* < 0.05; Fig. 3B). Under conventional conditions, the AG group also demonstrated numerically increased AFP relative to the BL group, though the adipogenic effect was less pronounced and not statistically significant (*P* > 0.05; Fig. 3B). These findings suggest that *A. communis* gavage significantly promotes AFD in chickens, with the adipogenic effects being partially mitigated under conventional conditions. Histological analysis of adipose tissue revealed that the average adipocyte area, diameter, and volume were significantly greater in the AGAb group than in the CNAb group (*P* < 0.05; Fig. 3C and D). Although the same parameters also showed numerical increases in the AG group compared with the BL group, these differences were not statistically significant (*P* > 0.05; Fig. 3C and D). No significant differences in adipocyte numbers were observed between the AGAb and CNAb groups or between the AG and BL groups (*P* > 0.05; Fig. 3C and D). Cecal histology further demonstrated that the AGAb group had significantly greater villus length and a higher average number of goblet cells per villus than the CNAb group (*P* < 0.05), while no significant differences were observed in other histological parameters (*P* > 0.05; Fig. 3E and F). In the AG group, villus mid-width and mucosal thickness were significantly reduced compared with the BL group (*P* < 0.05), with no significant differences in other parameters between the two groups (*P* > 0.05; Fig. 3E and F). Interestingly, other metrics, including villus length and goblet cell numbers per villus, showed similar numerical increases to those observed in the AGAb and CNAb groups, without reaching significance. Additionally, the villus surface area and villus length-to-crypt depth ratios showed a numerical increase following *A. communis* gavage in the gavage-treated and untreated groups (*P* > 0.05; Fig. 3E and F), although these differences were also not statistically significant. These results suggest that *A. communis* administration slightly enhances the digestive and absorptive capacity of the cecum, promoting nutrient assimilation that contributes to AFD primarily through adipocyte hypertrophy.

#### Impact of *A. communis* on cecal microbiome composition and function

**Fig. 4:**
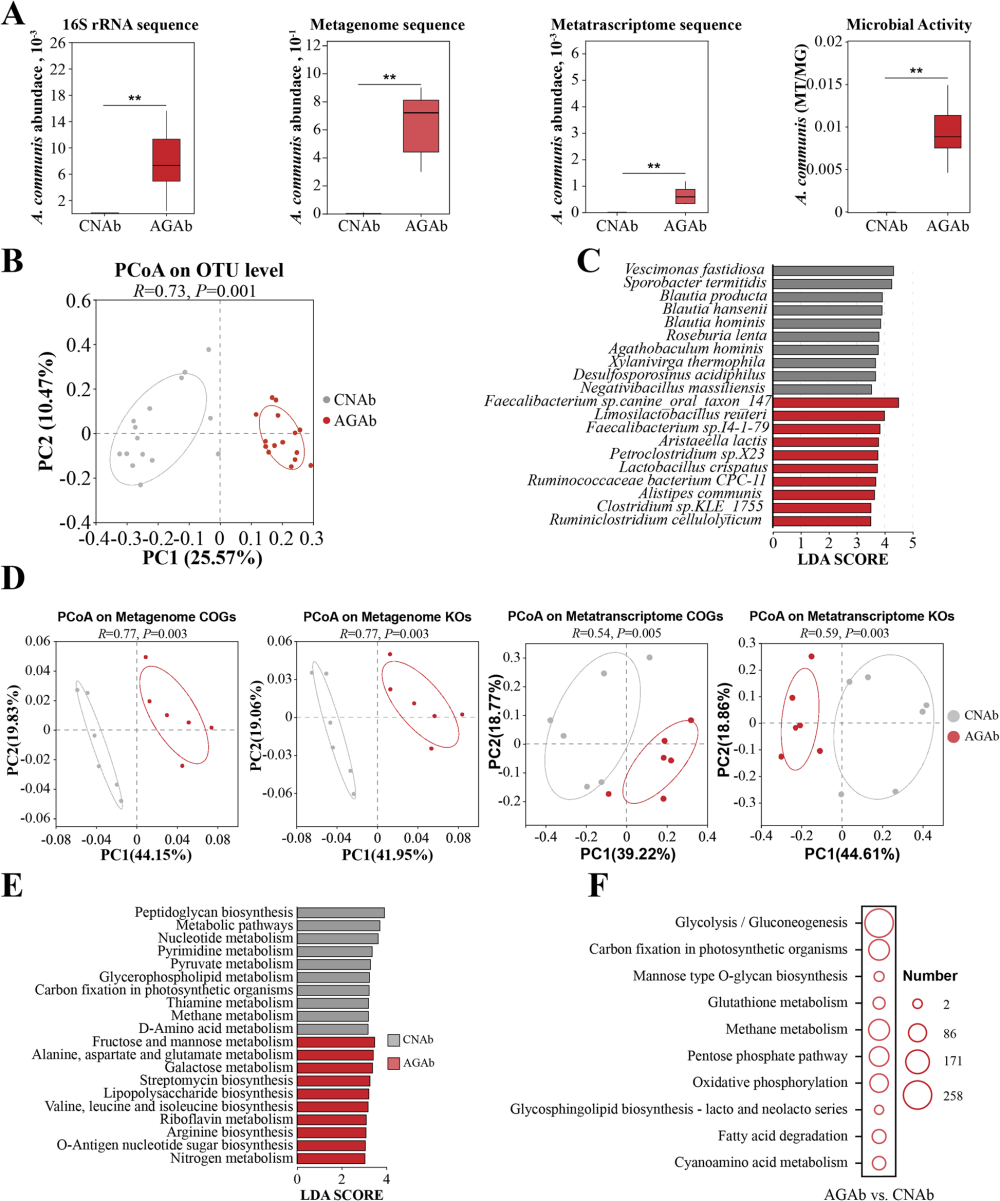
Comparative analysis of cecal taxonomic and functional composition in recipient chickens. **A** The comparison of relative abundance (left three panels) and activity (right panel) of *A. communis* between the AGAb and CNAb groups based on 16S rRNA (*n* = 15), MG (*n* = 6), and MT (*n* = 6) profiling; ^**^P < 0.01. **B** The PCoA of OTU levels for the AGAb and CNAb groups. **C** The comparative analysis of differential species between the AGAb and CNAb groups through LEfSE analysis; only the top 10 differential species are shown in the figure. **D** The PCoA of the cecal microbiome functional composition based on COG and KEGG KO profiles from MG (left two panels) and MT (right two panels) sequencing. **E** The comparison of KEGG pathways significantly enriched by differential microbial genes between the AGAb and CNAb groups in MG and MT profiling. LDA > 2 and *P* < 0.05 indicate significant difference between the comparisons; only the top 10 pathways are shown in the figure. **F** The KEGG pathway enrichments of differential expressed microbial genes in MT profiling between the AGAb and CNAb groups. CNAb, PBS control gavage with antibiotic pretreatment for 1 week post-hatch; AGAb, *A. communis* gavage with antibiotic pretreatment for 1 week post-hatch

Despite the observed effects on AFP, it remained unclear whether *A. communis* successfully colonized the cecum and was functionally active to exert these effects*.* 16S rRNA sequencing of cecal content in CNAb and AGAb chickens confirmed a significantly higher abundance of *A. communis* in the AGAb group than in the CNAb group (*P* < 0.05; Fig. 4A), which was further confirmed through MG and MT sequencing. Community composition analysis based on 16S rRNA sequencing data showed a distinct microbial profile in the AGAb group compared with the CNAb group (*P* < 0.05; Fig. 4B). Differential abundance analysis identified 126 microbial species with significant differences between the AGAb and CNAb groups (LDA score > 2.5, *P* < 0.05; Table S8). For instance, the AGAb group showed significantly increased abundances of *Faecalibacterium* sp. canine_oral_taxon_147, *Limosilactobacillus reuteri*, *Faecalibacterium* sp. I4-1-79, *Aristaeella lactis*, and *Lactobacillus crispatus*. By contrast, the abundances of *Vescimonas fastidiosa*, *Sporobacter termitidis*, *Blautia hansenii*, *Candidatus Gemmiger avium*, and *Aristaeella hokkaidonensis* were significantly decreased (LDA score > 2.5, *P* < 0.05; Fig. 4C; Table S8). These results demonstrate that *A. communis* successfully colonized the cecum of the recipient chickens and induced significant compositional shifts in the microbiota. PCoA based on COG IDs and KEGG KOs revealed significant functional separation between the AGAb and CNAb groups (Fig. 4D). Notably, microbial functions associated with carbohydrate and amino acid metabolism were significantly enriched and transcriptionally upregulated in the AGAb group (FDR < 0.05; Table S9; Fig. 4E and F). Taken together, these findings demonstrate that *A. communis* not only successfully colonized the cecum but also significantly modulated both the compositional and functional profiles of the gut microbiota, especially enhancing pathways related to amino acid and carbohydrate metabolism, thereby contributing to increased AFD in chickens.

#### Effects of *A. communis* on cecal microbiota metabolism

**Fig. 5:**
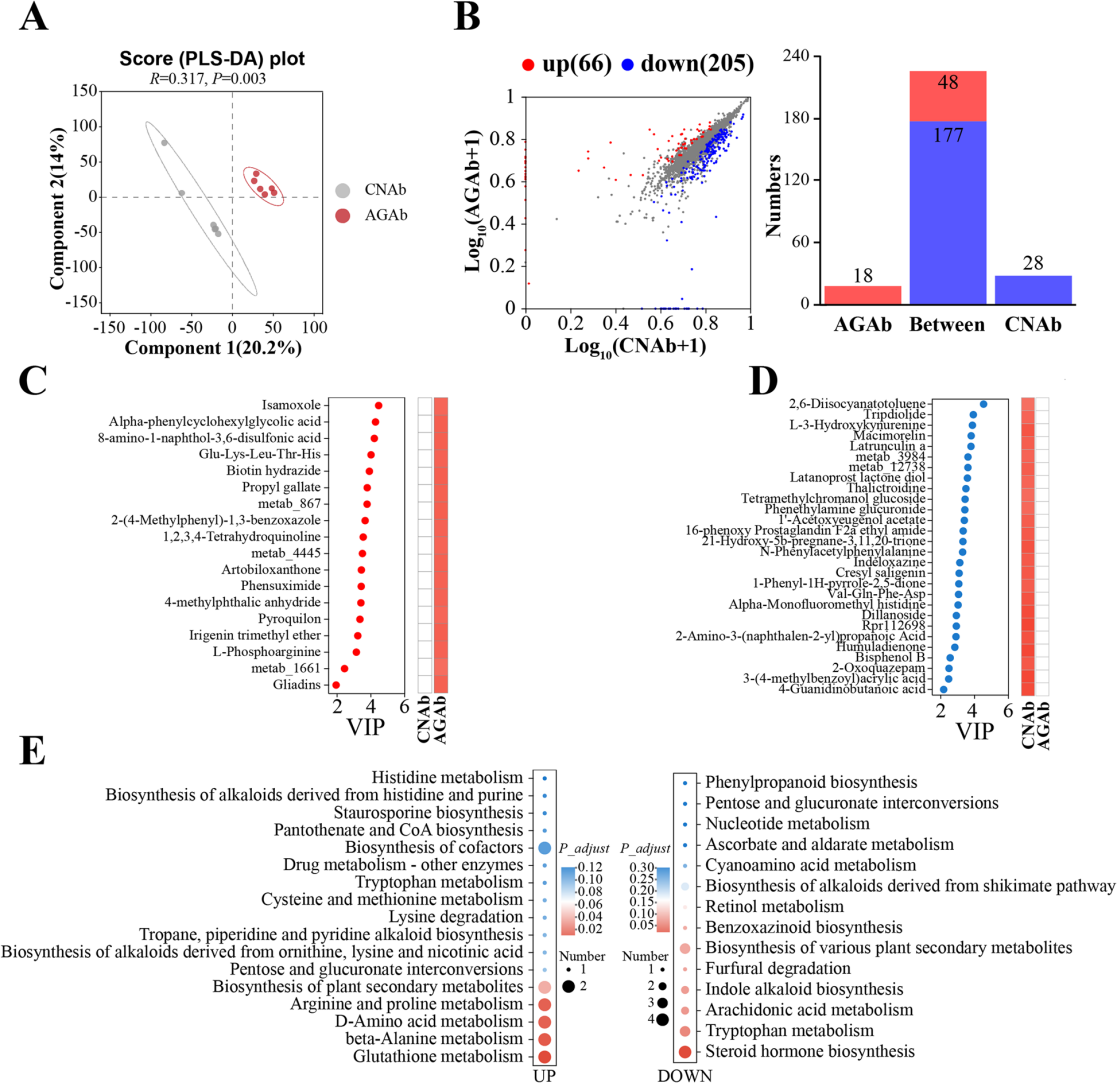
Comparative analysis of the cecal metabolome in recipient chickens. **A** Partial Least Squares Discriminant Analysis (PLS-DA) of the cecal metabolome composition in the AGAb and CNAb groups (*n* = 6). **B** The scatter plots of differential metabolite analysis; *x*- and *y*-axes represent expression levels in the two groups (log-transformed), and each point represents a specific metabolite. Red points represent significantly upregulated metabolites, blue points represent significantly downregulated metabolites, and gray points represent metabolites with no significant difference. The numbers of specifically expressed metabolites in each group are shown in the right part. **C** and **D** The VIP values and abundance of the specifically expressed metabolites in each group. metab_867, N-(2-benzoyl-3,4-dihydro-1H-isoquinolin-7-yl)thiophene-2-sulfonamide; metab_4445, [4-[2-(4-chlorophenyl)quinazolin-4-yl]piperazin-1-yl]-(furan-2-yl)methanone; metab_1661, 3-[4-Hydroxy-3-(3-methyl-2-butenyl)phenyl]-2-propenal; metab_3984, N-(4-ethoxyphenyl)-2-methyl-3-oxo-4H-pyrido [3,2-b][1,4]oxazine-2-carboxamide; metab_12738, 2-((4-Methoxy-3-methyl-2-pyridylmethyl) sulfo)-5-trifluoromethyl-1H-benzimidazole. **E** The KEGG pathway enrichment of significantly differential metabolites between the AGAb and CNAb groups. CNAb, PBS control gavage with antibiotic pretreatment for 1 week post-hatch; AGAb, *A. communis* gavage with antibiotic pretreatment for 1 week post-hatch

Metabolomic profiling of cecal contents from recipient chickens in the CNAb and AGAb groups revealed significant differences between the two groups (Fig. 5A). Differential analysis identified 271 differentially abundant metabolites (DAMs), with 66 upregulated and 205 downregulated in the AGAb group compared with the CNAb group (FDR < 0.05, VIP > 1, and FC > 1; Fig. 5B; Table S10). KEGG pathway enrichment analysis revealed that the upregulated DAMs were significantly enriched in glutathione, arginine, and proline metabolism (e.g., spermine and L-phosphoarginine), whereas downregulated DAMs were mainly involved in steroid hormone biosynthesis (e.g., 21-hydroxy-5b-pregnane-3,11,20-trione) (FDR < 0.05; Fig. 5E; Table S11). Notably, 18 DAMs were unique to the AGAb group, while 28 DAMs were exclusive to the CNAb group (Fig. 5B–D). The 18 AGAb group-specific DAMs were primarily classified as phenylpropanes, quinolones and derivatives, and amino acid derivatives, whereas the 28 CNAb group-specific DAMs included amino acids, peptides and analogs, and carbohydrate conjugates. These findings indicate that *A. communis* gavage reshapes amino acid and sterol metabolism in the cecal microbiome, consistent with the MG and MT profiling results.

#### Effects of A. communis on host serum metabolome

**Fig. 6:**
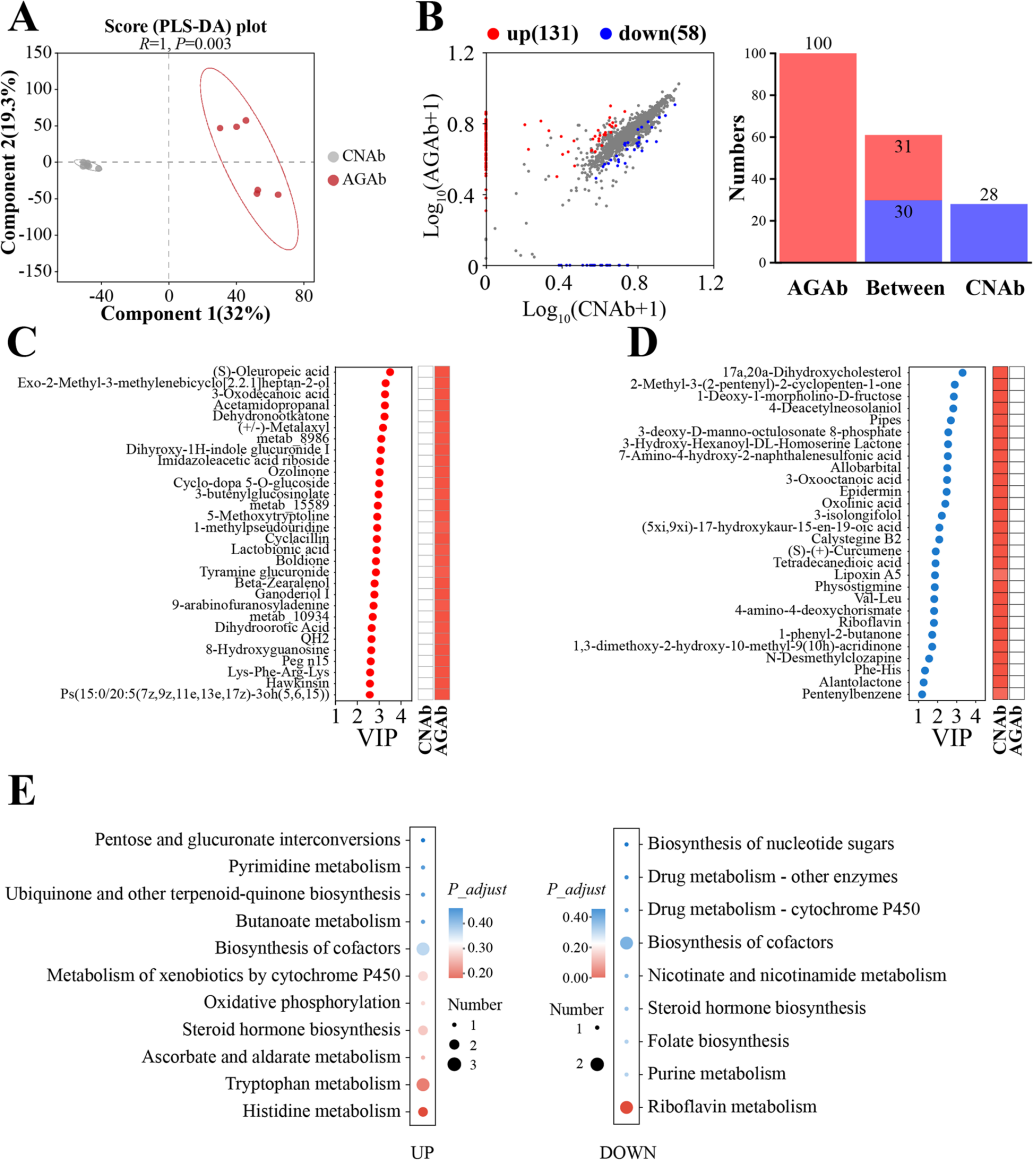
Comparative analysis of the serum metabolome in recipient chickens. **A** PLS-DA of the serum metabolome composition in the AGAb and CNAb groups (*n* = 6). **B** Scatter plots of differential metabolite analysis; x- and y-axes represent the expression levels in the two groups (log-transformed), and each point represents a specific metabolite. Red points represent significantly upregulated metabolites, blue points represent significantly downregulated metabolites, and gray points represent metabolites with no significant difference. The numbers of specifically expressed metabolites in each group are shown in the right part. **C** and **D** The Top30 VIP values and abundance of the specific expressed metabolites in each group. metab_8986, (1aalpha,2beta,3alpha,11calpha)-1a,2,3,11c-tetrahydro-6,11-dimethylbenzo[6,7]phenant-hro[3,4-b]oxirene-2,3-diol; metab_15589, 3-((4-(5-(hydroxymethyl)-2-oxo-3-oxazolidinyl)phenoxy) methyl)benzonitrile; metab_10934, 4-acetyl-3-hydroxy-2-propylphenyl (dimethylamino)methanethioate. **E** The KEGG pathway annotation of significantly differential metabolites between the AGAb and CNAb groups. CNAb, PBS control gavage with antibiotic pretreatment for 1 week post-hatch; AGAb, *A. communis* gavage with antibiotic pretreatment for 1 week post-hatch

Consistent with the cecal metabolic alterations, the serum metabolome exhibited significant separation between the AGAb and CNAb groups (Fig. 6A). A total of 189 DAMs were identified between the AGAb and CNAb groups, with 131 upregulated and 58 downregulated DAMs in the AGAb group (FDR < 0.05, VIP > 1 and FC > 1; Fig. 6B; Table S12). KEGG pathway enrichment analysis revealed that upregulated DAMs were mainly enriched in tryptophan and histidine metabolism (e.g., 3-(3-indolyl)-2-oxopropanoic acid, N-acetylhistamine, and imidazoleacetic acid riboside), whereas the downregulated DAMs were primarily associated with riboflavin metabolism (e.g., riboflavin) (Fig. 6E; Table S13). Interestingly, 100 DAMs were specific to the AGAb group, while only 28 DAMs were specific to the CNAb group (Table S12). Both sets were primarily annotated as amino acids, peptides and analogs, and carbohydrate conjugates. These results suggest that *A. communis* gavage substantially alters host amino acid and vitamin metabolism at the systemic level.

#### Effects of *A. communis* on gene expression in cecal mucosa, liver, and abdominal fat

**Fig. 7:**
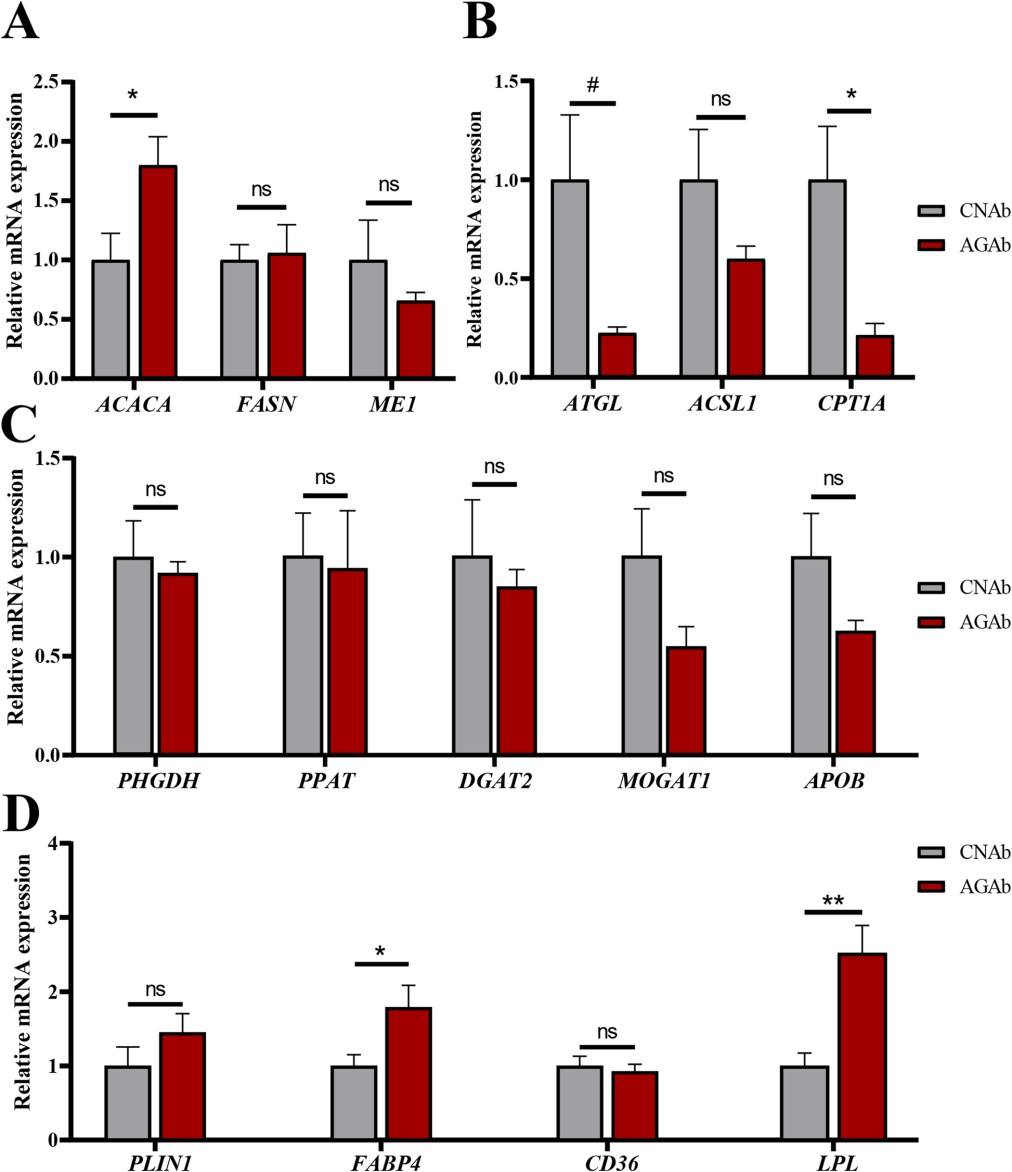
Effects of *A. communis* on lipid metabolism-related genes in the liver and abdominal fat. **A**–**C** The comparison of relative mRNA expressions of fatty acid synthesis-related maker genes in the liver (**A**) between the AGAb and CNAb groups (*n* = 6), as well as those involved in the lipolysis pathway (**B**) and triglyceride synthesis/transport (qPCR; **C**). **D** The comparison of relative mRNA expression of lipid deposition-associated genes in the abdominal fat tissue between the AGAb and CNAb groups (qPCR). CNAb, PBS control gavage with antibiotic pretreatment for 1 week post-hatch; AGAb, *A. communis* gavage with antibiotic pretreatment for 1 week post-hatch. # indicates *P* < 0.1, * indicates *P* < 0.05, ** indicates *P* < 0.01. ns means no significance

In aspect of host response, no significant differences in gene expression of cecal mucosa were observed between the AGAb and CNAb groups (Fig. S5), suggesting that *A. communis* does not exert major effects through transcriptional changes in the cecal mucosa. RT-qPCR was further used to compare the expression of key genes related to lipid metabolism in the liver (fatty acid synthesis, triglyceride synthesis/transport, and lipolysis) and abdominal fat tissue (lipid deposition) of AGAb and CNAb chickens. The results demonstrated that *A. communis* treatment resulted in enhanced hepatic fatty acid biosynthesis (upregulated *ACACA*) and suppressed lipolysis (downregulated *CPT1A*) (*P* < 0.05; Fig. 7A and B), leading to lipid accumulation in the liver, and enhanced fatty acid uptake/re-esterification in abdominal adipose tissue (elevated *FABP4* and *LPL*, *P* < 0.05; Fig. 7D).

**Fig. 8:**
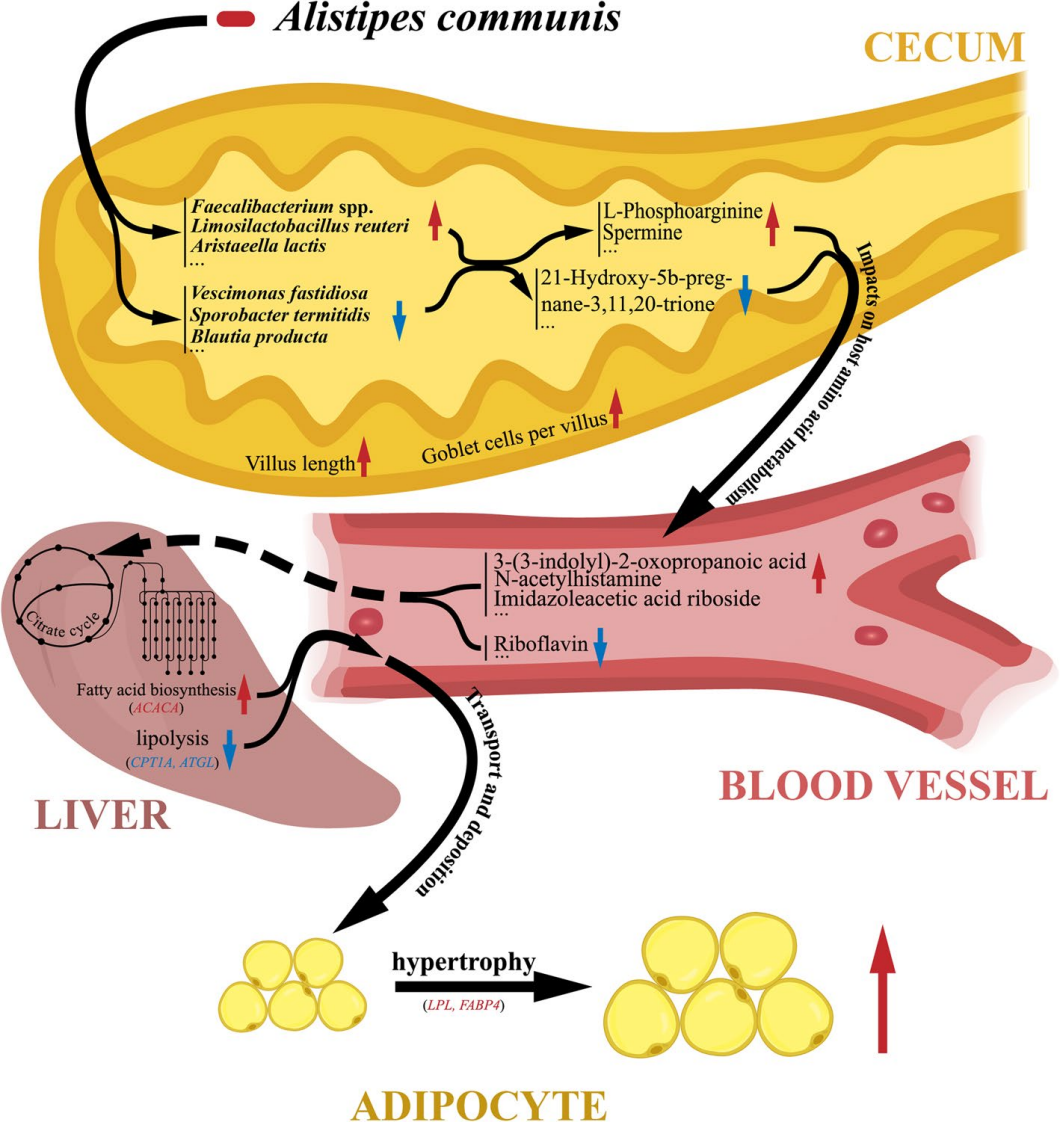
Regulatory mechanism of *A. communis* in AFD in chickens. Schematic of the potential mechanisms by which *A. communis* promotes AFD in chickens. *A. communis* gavage triggers a cascade of changes in the cecum, beginning with alterations in the microbiome composition and function. Specifically, it increases the abundances of *Faecalibacterium* spp., *Limosilactobacillus reuteri*, and *Aristaeella lactis*, while reducing the abundances of *Vescimonas fastidiosa*, *Sporobacter termitidis*, and *Blautia* spp. These microbiome shifts are accompanied by changes in the cecal metabolome, with an increase in L-phosphoarginine and spermine levels and a decrease in 21-hydroxy-5b-pregnane-3,11,20-trione levels. Subsequently, the altered cecal metabolome influences serum metabolic profiles in the host, increasing 3-(3-indolyl)-2-oxopropanoic acid, N-acetylhistamine, and imidazoleacetic acid riboside levels while decreasing riboflavin levels. This cascade ultimately promotes adipocyte hypertrophy and excessive abdominal fat deposition, possibly via TCA cycle-mediated fatty acid biosynthesis. The dotted line designates the predicted regulatory pathway based on priori information. The solid lines indicate the regulatory pathway of *A. communis* identified in our study. Red arrows indicate a significant increase, whereas blue arrows indicate a significant decrease. Gene names in red and blue represent upregulated and downregulated expression, respectively

In summary, this study provides the first evidence establishing a causal link between the cecal microbiota and chickens AFD through CMT experiments. Integrated MG and MT sequencing analyses identified *A. communis* as a dominant, active contributor to AFD in genetically obese chicken models. Subsequently, in vivo gavage experiments, combined with multi-omics profiling and qPCR, demonstrated that *A. communis* alters the cecal metabolome (e.g., L-phosphoarginine and spermine), disrupts systemic tryptophan and histamine metabolism (e.g., altering levels of serum 3-(3-indolyl)-2-oxopropanoic acid, N-acetylhistamine, and imidazoleacetic acid riboside) and induces metabolic perturbations that stimulate hepatic fatty acid biosynthesis while suppressing lipolysis. These changes culminate in adipocyte hypertrophy and AFD increase in chickens (Fig. 8).

## Discussion

The chicken cecal microbiome is characterized by high microbiability and has been implicated in lipid metabolism [10, 18, 19, 22]. Despite these findings, most studies have relied on correlation-based analyses from omics sequencing data, leaving the causal relationships between specific cecal microbes and AFD unresolved. In this study, we used genetic obese broiler model [23] and employed a comprehensive approach—combining CMT, multi-omics analyses, and in vivo gavage experiments—to identify key cecal microbes and their regulatory mechanisms in broiler AFD. Our results demonstrate that *A. communis* is a crucial microbial contributor to AFD, acting by modulating host tryptophan and histamine metabolism. These alterations in amino acid metabolism were associated with adipocyte hypertrophy, ultimately promoting excessive AFD in broilers. To our knowledge, this is the first study to establish a causal role of the cecal microbiome in AFD using microbiota transplantation in genetically lean and obese chickens. Furthermore, we precisely identified and validated the role of a specific cecal microbe in modulating AFD by conducting integrated host-microbiome data analyses and in vivo experiments. These findings provide novel insights into the gut microbiome’s role in avian obesity and suggest potential for advancing our understanding of obesity in mammals.

Causal links between the gut microbiota and obesity have been extensively reported in mammals. Transfer of gut microbiota from obese or lean donors replicates corresponding phenotypes in recipients and modulation of gut microbiota in obese mice alters obesity-related outcomes, underscoring the microbiome’s causal role and therapeutic potential in obesity [34–36]. Consistently, our results confirmed that cecal microbiota significantly influenced AFD phenotypes in chickens (Fig. 1C). Studies have shown that chicken cecal microbiota can be successfully transplanted via oral gavage, influencing microbiota development trajectory and exhibiting preferential colonization of homologous gut regions in recipients [37, 38]. In present study, although the transplanted microbiota altered the recipient communities in a donor-type-dependent manner (Fig. 1D and E), the compositions did not fully converge with those of the donors. This discrepancy may reflect incomplete transfer—due to loss of anaerobic microbes during gavage—or the influence of host genetics and physiology on microbiota colonization [39]. These findings confirm the efficacy of oral gavage as a robust protocol for delivering cecal microbiota in CMT. Collectively, our CMT experiments support the conclusion that differences in the cecal microbiome drive divergent AFD phenotypes.

Emerging evidence suggests that changes in microbial gene expression (MT data) often exceed those in DNA-based taxonomic composition (MG data), highlighting functional divergences otherwise undetectable through DNA-based methods [40, 41]. Consistently, our MT sequencing profiles revealed more differentially abundant species between the fat- and lean-line broilers than MG data (Fig. 2A–C). Integrative MG–MT approaches enable a multi-dimensional characterization of gut microbial ecosystems and enhance resolution [41, 42]. By correlating transcriptional activity (RNA/DNA ratios) with clinical phenotypic traits, researchers have pinpointed disease-driving species such as *Streptococcus mutans* (caries) and *Porphyromonas gingivalis* (periodontitis) as metabolically dominant pathobionts [43]. Thus, we calculated RNA/DNA ratios and their correlations with AFD traits, identifying eight core species (Fig. 2C and D). Strikingly, *A. communis* demonstrated significantly elevated microbial activity in fat line and strong positive correlations with AFP and AFW (Fig. 2C and D), identifying this bacterium as a key functional modulator of AFD. Moreover, the MT data revealed significant differences in the relative abundances of several *Faecalibacterium* species between the fat and lean lines (Fig. 2B). We also observed significantly elevated levels of several *Faecalibacterium* species in the *A. communis* gavage group (Fig. 4C). *Faecalibacterium*, which engages in complex regulatory interactions with a variety of gut microorganisms, can have its levels selectively increased by specific microbes [44]. Research indicates that specific compounds synthesized by one bacterial species can facilitate the growth of others by providing growth substrates, a process termed cross-feeding [45]. Therefore, we propose that *Faecalibacterium* species and *A. communis* may potentially involve cross feeding interactions. However, most research reveals a significant negative correlation between *Faecalibacterium* and fat deposition [46–48]. This is attributed to its production of butyrate, which reduces liver and abdominal fat by activating the AMPK signaling pathway and stimulating fatty acid oxidation [46–48]. Although butyrate is generally considered an important factor that mitigates fat deposition, it can also inhibit histone deacetylase activity, thereby promoting the expression of genes that drive adipocyte differentiation [49, 50]. Notably, our data revealed that *A. communis* gavage significantly increased serum butyric acid levels (Table S12), which is consistent with the observed increase in *Faecalibacterium* species. Collectively, we speculate that the increase in *Faecalibacterium* species following *A. communis* gavage may promote adipocyte differentiation through their production of butyrate, acting synergistically with *A. communis* to drive AFD.

Although germ-free and gnotobiotic models are powerful tools for studying host–microbe interplay [51], they are resource-intensive. Pretreatment with broad-spectrum antibiotics offers a practical alternative by rapidly depleting indigenous microbiota before microbial inoculation [52]. This strategy has been employed to develop pseudo-germ-free animals and assess the role of specific microbes, such as *Blautia producta*, in lipid metabolism [53], as well as to uncover microbial contributions to diabetic cardiomyopathy [54]. In this study, applying this strategy, we confirmed the pro-adipogenic role of *A. communis* through significantly increased AFP and adipocyte size in the antibiotic-treated AGAb group (Fig. 3B–D). Although our initial attempts to directly isolate and culture the target *A. communis* from the donor cecum did not yield viable isolates, we proceeded with the commercial strain *A. communis* 5CBH24 (DSM: 108979) to assess its role in modulating host lipid metabolism. This alternative approach is justified by the principle that core metabolic functions are often conserved within a bacterial species, despite strain-level variations [28]. Furthermore, it is supported by precedent studies, such as that of Xie et al., which successfully utilized commercial strains to investigate the role of gut microbes in regulating intramuscular fat deposition [55]. In *A. communis* gavage experiments, no statistically significant differences were observed between the AG and BL groups following *A. communis* gavage under conventional conditions. Current ecological theory posits that microbial community dynamics are governed by two complementary mechanisms: equalizing effects that minimize fitness disparities between species and stabilizing effects mediated through niche differentiation (e.g., spatial resource partitioning or metabolic specialization) [56]. Therefore, we inferred that *A. communis* gavage exerted a limited influence on the complex, niche-structured metabolic networks sustained by the other indigenous microbiota under conventional conditions.

As a newly discovered species within *Alistipes* [57], the mechanistic contributions of *A. communis* to its host remains unexplored, with no studies to date elucidating its functional role in physiological processes. Gut-resident *Alistipes* influences host tryptophan metabolism, ameliorating depression and anxiety-like behaviors [58], while its abundance is affected by dietary factors like isoleucine, possibly through negative feedback mechanisms [59]. These findings underscore the relationship between *Alistipes* species and amino acid metabolism. In our study, metabolomic analysis following *A. communis* colonization revealed significantly elevated levels of specific metabolites: L-phosphoarginine, spermine, and 5-(2′-carboxyethyl)-4,6-dihydroxypicolinate in the cecum, and N-acetylhistamine, imidazoleacetic acid riboside, 3,4-dihydroxyhydrocinnamic acid, 6-hydroxymelatonin, and 3-(3-indolyl)-2-oxopropanoic acid in the serum (Figs. 5 and 6; Tables S10 and S12). Arginine, a semi-essential amino acid, is the primary precursor for nitrogen-containing metabolites, including polyamine, ornithine, and proline [60]. Notably, arginine and histidine can be interconverted via glutamate within KEGG metabolism pathways (https://www.genome.jp/dbget-bin/www_bget?C00025). Spermine supplementation in rats elevates serum amino acids (e.g., 1-methylhistidine), potentially due to the conversion of exogenous spermine into serum aldehydes followed by aldehyde dehydrogenase-mediated transfer to other amino acids [61, 62]. The compound 5-(2'-carboxyethyl)-4,6-dihydroxypicolinate in the cecum is involved in the tryptophan metabolism pathway, as 3,4-dihydroxyhydrocinnamic acid, 6-hydroxymelatonin, and 3-(3-indolyl)-2-oxopropanoic acid in serum (Tables S10 and S12). These findings suggest that *A. communis*-induced increases in arginine, spermine, and tryptophan derivatives in the cecum can be converted into corresponding amino acid metabolites in the host circulatory system, particularly histidine, tryptophan, and their derivatives, thereby affecting host amino acid metabolism. Interestingly, the AGAb group exhibited significantly greater villus length and goblet cell counts per villus than the CNAb group. And numerical increases in villus area and villus length-to-crypt depth ratios were also noted (Fig. 3E and F). The histological structure of the intestine, particularly increased numbers of goblet cells, larger villus areas, and shallower crypts, directly impacts its digestive and absorptive functions by augmenting enzyme secretion, digestion, and nutrient absorption and transport within the intestine [63, 64]. Therefore, our findings suggest that *A. communis* can subtly improve the absorption and transport of these metabolites from the cecal lumen into the host serum. Collectively, these findings underscore that *Alistipes* species is critically involved in directly or indirectly regulating host amino acid metabolism, thus reinforcing their functional significance in the host–microbe metabolic crosstalk in obesity. Recent research highlights the significant role of amino acids in hepatic lipid metabolism, both as a major carbon source for the TCA cycle and lipogenesis in mouse hepatocytes and as a more efficient fuel for fatty acid synthesis compared to glucose [65]. Emerging evidence reveals that reduced circulating levels of serine and glycine are strongly correlated with metabolic disturbances including obesity, type 2 diabetes, and dyslipidemia, which positions amino acid metabolism as a critical regulatory pathway in the development of adiposity-related pathologies [66]. Similarly, our study demonstrated that orally administered *A. communis* increases the levels of amino acids and their derivatives in both the cecum and serum, promoting increased AFD in chickens. Meanwhile, gene expression analysis showed that the expression of a fatty acid synthesis-related marker gene (*ACACA*) was significantly upregulated in the liver after *A. communis* gavage (Fig. 7A). Therefore, we suspect that *A. communis* gavage disrupts host amino acid homeostasis, potentially redirecting excess carbon skeletons from aberrant amino acid catabolism into the TCA cycle in the liver. Specifically, whether these effects are mediated through a redirection of amino acid carbon flux toward de novo lipogenesis driven by the TCA cycle remain unclear. Therefore, further experimental validation is warranted to address these uncertainties.

## Conclusions

In summary, our study demonstrates that *A. communis* gavage significantly alters amino acid metabolism and putrefaction in the cecum (e.g., L-phosphoarginine, spermine, and 5-(2′-carboxyethyl)-4,6-dihydroxypicolinate), leading to increased serum levels of tryptophan and histidine metabolic derivatives in the host (e.g., 2-amino-3-methoxybenzoic acid, N-acetylhistamine, imidazoleacetic acid riboside, and 6-hydroxymelatonin). These variations ultimately contribute to augmented AFD in chickens through adipocyte hypertrophy. These effects may potentially be driven by fatty acid de novo biosynthesis in the liver (*ACACA*), fatty acid uptake, and re-esterification in adipose tissue (*LPL* and *FABP4*) (Fig. 8). Our study thus offers valuable insights by identifying a cecal microbial factor promoting AFD in broilers and provides new perspectives on understanding the regulatory mechanisms of the gut microbiome underlying host obesity.

## Supplementary Information

Additional file 1: Table S1. Identification of *Alistipes communis* suspension by 16S rRNA gene sequencing. Table S2. The specific primers for lipid metabolism related marker genes in qPCR. Table S3. The abundance matrix of cecal genus in recipient AA chickens. Table S4. The abundance matrix of cecal phylum in recipient AA chickens. Table S5. Metagenomic sequencing data statistics. Table S6. Metatranscriptome sequencing data statistics. Table S7. The LEfSe analysis of differential abundence species between the two lines’ donors. Table S8. Significantly changed species in the recipient cecum after *Alistipes communis* administration based on 16S sequencing. Table S9. Significantly changed KEGG kos in the recipient cecum after *Alistipes communis* administration based on metagenome and metatranscriptome sequencing. Table S10. Significantly changed cecal metabolites in the recipient cecum after *Alistipes communis* administration. Table S11. The KEGG enricment of the significantly changed cecal metabolites. Table S12. Significantly changed serum metabolites in the recipient cecum after *Alistipes communis* administration. Table S13. The KEGG enricment of the significantly changed host serum metabolites.

Additional file 2: Fig. S1 Comparative analysis of cecal microbial diversity and composition between donor and recipient broiler chickens in the cecal microbiota transplantation experiment. Fig. S2 Cecal microbial composition of experimental donor broiler chickens in the metagenome. Fig. S3 Comparative analysis of cecal microbiota composition in experimental donor broiler chickens from metatranscriptomic data. Fig. S4 The microbial diversity comparison after antibiotics treatments. Fig. S5 Differential gene expression analysis of cecal mucosa in recipient broiler chickens following *Alistipes communis* transplantation.

## Data Availability

All sequencing data generated this study are deposited in the Sequence Read Archive and the corresponding BioProject accession number is PRJNA1260715 (https://dataview.ncbi.nlm.nih.gov/object/PRJNA1260715?reviewer=n7tfhlldb6cbmvojf9pb7ivj5a). All raw metabolome data generated in this study are available in EMBI-EBI MetaboLights database with the identifier MTBLS12720 (https://www.ebi.ac.uk/metabolights/reviewer252bca82-fa93-4cbd-9851-c28ea2e100d2).

## Abbreviations

AFD: Abdominal fat deposition
AFP: Abdominal fat percentage
AFW: Abdominal fat weight
AG: *A. communis* gavage without antibiotic pretreatment
AGAb: *A. communis* gavage with antibiotic pretreatment for 1 week post-hatch
BL: Blank, without antibiotics pretreatments
BW: Body weight
CMT: Cecal microbiota transplant
CNAb: PBS control gavage with antibiotic pretreatment for 1 week post-hatch
CP: Crude protein
FLA: Gavage group with fat-line donor cecal microbiota suspension
H&E: Hematoxylin and eosin
LLA: Gavage group with lean-line donor cecal microbiota suspension
LPL: Lipoprotein lipase
ME: Metabolizable energy
MG: Metagenomic
MT: Metatranscriptomic
NEAUHLF: The Northeast Agricultural University broiler lines divergently selected for abdominal fat content
SCFAs: Short-chain fatty acids
TMAO: Trimethylamine N-oxide

## References

[1] Rosenberg E, Zilber-Rosenberg I. The hologenome concept of evolution after 10 years. Microbiome. 2018;6:78. 10.1186/s40168-018-0457-9. 29695294 10.1186/s40168-018-0457-9 PMC5922317

[2] Hoyles L, Fernández-Real J-M, Federici M, Serino M, Abbott J, Charpentier J, et al. Molecular phenomics and metagenomics of hepatic steatosis in non-diabetic obese women. Nat Med. 2018;24:1628. 10.1038/s41591-018-0169-5. 29942096 10.1038/s41591-018-0061-3 PMC6140997

[3] Thackray VG. Sex, microbes, and polycystic ovary syndrome. Trends Endocrinol Metab. 2019;30(1):54–65. 10.1016/j.tem.2018.11.001. 30503354 10.1016/j.tem.2018.11.001 PMC6309599

[4] Zhao S, Lieberman TD, Poyet M, Kauffman KM, Gibbons SM, Groussin M, et al. Adaptive evolution within gut microbiomes of healthy people. Cell Host Microbe. 2019;25(5):656–67.e8. 10.1016/j.chom.2019.03.007. 31028005 10.1016/j.chom.2019.03.007 PMC6749991

[5] Gomes AC, Hoffmann C, Mota JF. The human gut microbiota: metabolism and perspective in obesity. Gut Microbes. 2018;9(4):308–25. 10.1080/19490976.2018.1465157. 29667480 10.1080/19490976.2018.1465157 PMC6219651

[6] Canfora EE, Meex RC, Venema K, Blaak EE. Gut microbial metabolites in obesity, NAFLD and T2DM. Nat Rev Endocrinol. 2019;15:261–73. 10.1038/s41574-019-0156-z. 30670819 10.1038/s41574-019-0156-z

[7] Chanda D, De D. Meta-analysis reveals obesity associated gut microbial alteration patterns and reproducible contributors of functional shift. Gut Microbes. 2024;16(1):2304900. 10.1080/19490976.2024.2304900. 38265338 10.1080/19490976.2024.2304900 PMC10810176

[8] Wu C, Yang F, Zhong H, Hong J, Lin H, Zong M, et al. Obesity-enriched gut microbe degrades myo-inositol and promotes lipid absorption. Cell Host Microbe. 2024;32(8):1301–14.e9. 10.1016/j.chom.2024.06.012. 38996548 10.1016/j.chom.2024.06.012

[9] Chen C, Fang S, Wei H, He M, Fu H, Xiong X, et al. *Prevotellacopri* increases fat accumulation in pigs fed with formula diets. Microbiome. 2021;9:175. 10.1186/s40168-021-01110-0. 34419147 10.1186/s40168-021-01110-0 PMC8380364

[10] Chen Y, Akhtar M, Ma Z, Hu T, Liu Q, Pan H, et al. Chicken cecal microbiota reduces abdominal fat deposition by regulating fat metabolism. NPJ Biofilms Microbiomes. 2023;9:28. 10.1038/s41522-023-00390-8. 37253749 10.1038/s41522-023-00390-8 PMC10229630

[11] Turnbaugh PJ, Hamady M, Yatsunenko T, Cantarel BL, Duncan A, Ley RE, et al. A core gut microbiome in obese and lean twins. Nature. 2009;457:480–4. 10.1038/nature07540. 19043404 10.1038/nature07540 PMC2677729

[12] von Engelhardt W, Bartels J, Kirschberger S, Meyer zu Düttingdorf HD, Busche R. Role of short-chain fatty acids in the hind gut. Vet Q. 1998;20(Sup3):52–9. 10.1080/01652176.1998.9694970. 9689727

[13] Barnes EM. The avian intestinal flora with particular reference to the possible ecological significance of the cecal anaerobic bacteria. Am J Clin Nutr. 1972;25(12):1475–9. 10.1093/ajcn/25.12.1475. 4346128 10.1093/ajcn/25.12.1475

[14] Bäckhed F, Ding H, Wang T, Hooper LV, Koh GY, Nagy A, et al. The gut microbiota as an environmental factor that regulates fat storage. Proc Natl Acad Sci U S A. 2004;101(44):15718–23. 10.1073/pnas.0407076101. 15505215 10.1073/pnas.0407076101 PMC524219

[15] Wen Y, Luo Y, Qiu H, Chen B, Huang J, Lv S, et al. Gut microbiota affects obesity susceptibility in mice through gut metabolites. Front Microbiol. 2024;15:1343511. 10.3389/fmicb.2024.1343511. 38450171 10.3389/fmicb.2024.1343511 PMC10916699

[16] Fu T, Huan T, Rahman G, Zhi H, Xu Z, Oh TG, et al. Paired microbiome and metabolome analyses associate bile acid changes with colorectal cancer progression. Cell Rep. 2023;42(8):112997. 10.1016/j.celrep.2023.112997. 10.1016/j.celrep.2023.112997 PMC10903535 37611587

[17] Wang Y, Zhou P, Zhou X, Fu M, Wang T, Liu Z, et al. Effect of host genetics and gut microbiome on fat deposition traits in pigs. Front Microbiol. 2022;13:925200. 10.3389/fmicb.2022.925200. 36204621 10.3389/fmicb.2022.925200 PMC9530793

[18] Wen C, Yan W, Sun C, Ji C, Zhou Q, Zhang D, et al. The gut microbiota is largely independent of host genetics in regulating fat deposition in chickens. ISME J. 2019;13(6):1422–36. 10.1038/s41396-019-0367-2. 30728470 10.1038/s41396-019-0367-2 PMC6775986

[19] Xiang H, Gan J, Zeng D, Li J, Yu H, Zhao H, et al. Specific microbial taxa and functional capacity contribute to chicken abdominal fat deposition. Front Microbiol. 2021;12:643025. 10.3389/fmicb.2021.643025. 33815329 10.3389/fmicb.2021.643025 PMC8010200

[20] Jing Y, Yuan Y, Monson M, Wang P, Mu F, Zhang Q, et al. Multi-omics association reveals the effects of intestinal microbiome–host interactions on fat deposition in broilers. Front Microbiol. 2022;12:815538. 10.3389/fmicb.2021.815538. 35250914 10.3389/fmicb.2021.815538 PMC8892104

[21] Zerehdaran S, Vereijken AJ, Van Arendonk J, Van der Waaijt E. Estimation of genetic parameters for fat deposition and carcass traits in broilers. Poult Sci. 2004;83(4):521–5. 10.1093/ps/83.4.521. 15109049 10.1093/ps/83.4.521

[22] Yin Z, Ji S, Yang J, Guo W, Li Y, Ren Z, et al. Cecal microbial succession and its apparent association with nutrient metabolism in broiler chickens. mSphere. 2023;8:e00614-22. 10.1128/msphere.00614-22. 37017520 10.1128/msphere.00614-22 PMC10286727

[23] Guo L, Sun B, Shang Z, Leng L, Wang Y, Wang N, et al. Comparison of adipose tissue cellularity in chicken lines divergently selected for fatness. Poult Sci. 2011;90(9):2024–34. 10.3382/ps.2010-00863. 21844269 10.3382/ps.2010-00863

[24] National Research Council. Nutrient requirements of poultry. 9th ed. Washington DC: The National Academies; 1994.

[25] Hu J, Chen L, Tang Y, Xie C, Xu B, Shi M, et al. Standardized preparation for fecal microbiota transplantation in pigs. Front Microbiol. 2018;9:1328. 10.3389/fmicb.2018.01328. 29971061 10.3389/fmicb.2018.01328 PMC6018536

[26] Abbas W, Bi R, Hussain MD, Tajdar A, Guo F, Guo Y, et al. Antibiotic cocktail effects on intestinal microbial community, barrier function, and immune function in early broiler chickens. Antibiotics Basel. 2024;13(5):413. 10.3390/antibiotics13050413. 38786141 10.3390/antibiotics13050413 PMC11117290

[27] Yang J, Pei G, Sun X, Xiao Y, Miao C, Zhou L, et al. RhoB affects colitis through modulating cell signaling and intestinal microbiome. Microbiome. 2022;10:149. 10.1186/s40168-022-01347-3. 36114582 10.1186/s40168-022-01347-3 PMC9482252

[28] Mukhopadhya I, Martin JC, Shaw S, Gutierrez-Torrejon M, Boteva N, McKinley AJ, et al. Novel insights into carbohydrate utilisation, antimicrobial resistance, and sporulation potential in *Roseburia intestinalis* isolates across diverse geographical locations. Gut Microbes. 2025;17(1):2473516. 10.1080/19490976.2025.2473516. 40089923 10.1080/19490976.2025.2473516 PMC11913394

[29] Rodig SJ. Preparing paraffin tissue sections for staining. Cold Spring Harb Protoc. 2021;2021(3):pdb. prot099663. 10.1101/pdb.prot099663. 10.1101/pdb.prot099663 33649119

[30] Goldrick RB. Morphological changes in the adipocyte during fat deposition and mobilization. Am J Physiol. 1967;212(4):777–82. 10.1152/ajplegacy.1967.212.4.777. 6024438 10.1152/ajplegacy.1967.212.4.777

[31] Björntorp P, Sjöström L. Number and size of adipose tissue fat cells in relation to metabolism in human obesity. Metabolism. 1971;20(7):703–13. 10.1016/0026-0495(71)90084-9. 5090134 10.1016/0026-0495(71)90084-9

[32] Xie Z, Bai Y, Chen G, Rui Y, Chen D, Sun Y, et al. Modulation of gut homeostasis by exopolysaccharides from *Aspergillus cristatus* (MK346334), a strain of fungus isolated from Fuzhuan brick tea, contributes to immunomodulatory activity in cyclophosphamide-treated mice. Food Funct. 2020;11(12):10397–412. 10.1039/d0fo02272a. 33237077 10.1039/d0fo02272a

[33] Broeders S, Huber I, Grohmann L, Berben G, Taverniers I, Mazzara M, et al. Guidelines for validation of qualitative real-time PCR methods. Trends Food Sci Technol. 2014;37(2):115–26. 10.1016/j.tifs.2014.03.008.

[34] Bäckhed F, Manchester JK, Semenkovich CF, Gordon JI. Mechanisms underlying the resistance to diet-induced obesity in germ-free mice. Proc Natl Acad Sci U S A. 2007;104(3):979–84. 10.1073/pnas.0605374104. 17210919 10.1073/pnas.0605374104 PMC1764762

[35] Ridaura VK, Faith JJ, Rey FE, Cheng J, Duncan AE, Kau AL, et al. Gut microbiota from twins discordant for obesity modulate metabolism in mice. Science. 2013;341(6150):1241214. 10.1126/science.1241214. 24009397 10.1126/science.1241214 PMC3829625

[36] Chang CJ, Lin CS, Lu CC, Martel J, Ko YF, Ojcius DM, et al. *Ganoderma lucidum* reduces obesity in mice by modulating the composition of the gut microbiota. Nat Commun. 2015;6:7489. 10.1038/ncomms8489. 26102296 10.1038/ncomms8489 PMC4557287

[37] Glendinning L, Chintoan-Uta C, Stevens MP, Watson M. Effect of cecal microbiota transplantation between different broiler breeds on the chick flora in the first week of life. Poult Sci. 2022;101(2):101624. 10.1016/j.psj.2021.101624. 34936955 10.1016/j.psj.2021.101624 PMC8704443

[38] Li N, Zuo B, Huang S, Zeng B, Han D, Li T, et al. Spatial heterogeneity of bacterial colonization across different gut segments following inter-species microbiota transplantation. Microbiome. 2020;8:161. 10.1186/s40168-020-00917-7. 33208178 10.1186/s40168-020-00917-7 PMC7677849

[39] Kurilshikov A, Wijmenga C, Fu J, Zhernakova A. Host genetics and gut microbiome: challenges and perspectives. Trends Immunol. 2017;38(9):633–47. 10.1016/j.it.2017.06.003. 28669638 10.1016/j.it.2017.06.003

[40] McNulty NP, Yatsunenko T, Hsiao A, Faith JJ, Muegge BD, Goodman AL, et al. The impact of a consortium of fermented milk strains on the gut microbiome of gnotobiotic mice and monozygotic twins. Sci Transl Med. 2011;3(106):106ra106. 10.1126/scitranslmed.3002701. 22030749 10.1126/scitranslmed.3002701 PMC3303609

[41] Li F, Hitch TC, Chen Y, Creevey CJ, Guan LL. Comparative metagenomic and metatranscriptomic analyses reveal the breed effect on the rumen microbiome and its associations with feed efficiency in beef cattle. Microbiome. 2019;7:6. 10.1186/s40168-019-0618-5. 30642389 10.1186/s40168-019-0618-5 PMC6332916

[42] Heintz-Buschart A, May P, Laczny CC, Lebrun LA, Bellora C, Krishna A, et al. Integrated multi-omics of the human gut microbiome in a case study of familial type 1 diabetes. Nat Microbiol. 2016;2:16180. 10.1038/nmicrobiol.2016.180. 27723761 10.1038/nmicrobiol.2016.180

[43] Belstrøm D, Constancias F, Liu Y, Yang L, Drautz-Moses DI, Schuster SC, et al. Metagenomic and metatranscriptomic analysis of saliva reveals disease-associated microbiota in patients with periodontitis and dental caries. NPJ Biofilms Microbiomes. 2017;3:23. 10.1038/s41522-017-0031-4. 28979798 10.1038/s41522-017-0031-4 PMC5624903

[44] Negishi H, Ichikawa A, Takahashi S, Kano H, Makino S. Targeted prebiotic application of gluconic acid-containing oligosaccharides promotes *Faecalibacterium* growth through microbial cross-feeding networks. ISME J. 2025;19(1):wraf027. 10.1093/ismejo/wraf027. 39936592 10.1093/ismejo/wraf027 PMC11922316

[45] Zhou Z, Jiang A, Jiang X, Hatzios SK. Metabolic cross-feeding of a dietary antioxidant enhances anaerobic energy metabolism by human gut bacteria. Cell Host Microbe. 2025;33(8):1321-32.E9. 10.1016/j.chom.2025.07.008. 40763732 10.1016/j.chom.2025.07.008 PMC12683948

[46] Munukka E, Rintala A, Toivonen R, Nylund M, Yang B, Takanen A, et al. *Faecalibacterium prausnitzii* treatment improves hepatic health and reduces adipose tissue inflammation in high-fat fed mice. ISME J. 2017;11(7):1667–79. 10.1038/ismej.2017.24. 28375212 10.1038/ismej.2017.24 PMC5520144

[47] Iino C, Endo T, Mikami K, Hasegawa T, Kimura M, Sawada N, et al. Significant decrease in Faecalibacterium among gut microbiota in nonalcoholic fatty liver disease: a large BMI-and sex-matched population study. Hepatol Int. 2021;13:748–56. 10.1007/s12072-019-09987-8. 10.1007/s12072-019-09987-8 31515740

[48] Martín R, Rios-Covian D, Huillet E, Auger S, Khazaal S, Bermúdez-Humarán LG, et al. *Faecalibacterium*: a bacterial genus with promising human health applications. FEMS Microbiol Rev. 2023;47(4):fuad039. 10.1093/femsre/fuad039. 37451743 10.1093/femsre/fuad039 PMC10410495

[49] Singh K, Koroma AK, Pandey RK, Wang Y, Cheng J, Haas P, et al. Restoring histone acetylation accelerates diabetic wound repair by improving the spatiotemporal dynamics of macrophages. Adv Sci (Weinh). 2025;12(46):e04920. 10.1002/advs.202504920. 10.1002/advs.202504920 PMC12697871 41051359

[50] Zhang L, Liu C, Jiang Q, Yin Y. Butyrate in energy metabolism: there is still more to learn. Trends Endocrinol Metab. 2021;32(3):159–69. 10.1016/j.tem.2020.12.003. 33461886 10.1016/j.tem.2020.12.003

[51] Kubelkova K, Benuchova M, Kozakova H, Sinkora M, Krocova Z, Pejchal J, et al. Gnotobiotic mouse model’s contribution to understanding host–pathogen interactions. Cell Mol Life Sci. 2016;73(20):3961–9. 10.1007/s00018-016-2341-8. 27544211 10.1007/s00018-016-2341-8 PMC11108488

[52] Lundberg R, Toft MF, August B, Hansen AK, Hansen CH. Antibiotic-treated versus germ-free rodents for microbiota transplantation studies. Gut Microbes. 2016;7(1):68–74. 10.1080/19490976.2015.1127463. 26744774 10.1080/19490976.2015.1127463 PMC4856451

[53] Xu W, Yu J, Yang Y, Li Z, Zhang Y, Zhang F, et al. Strain-level screening of human gut microbes identifies Blautia producta as a new anti-hyperlipidemic probiotic. Gut microbes. 2023;15(1):2228045. 10.1080/19490976.2023.2228045. 37408362 10.1080/19490976.2023.2228045 PMC10324434

[54] Zheng H, Zhang X, Li C, Wang D, Shen Y, Lu J, et al. BCAA mediated microbiota-liver-heart crosstalk regulates diabetic cardiomyopathy via FGF21. Microbiome. 2024;12:157. 10.1186/s40168-024-01872-3. 39182099 10.1186/s40168-024-01872-3 PMC11344321

[55] Xie C, Teng J, Wang X, Xu B, Niu Y, Ma L, et al. Multi-omics analysis reveals gut microbiota-induced intramuscular fat deposition via regulating expression of lipogenesis-associated genes. Anim Nutr. 2022;9:84–99. 10.1016/j.aninu.2021.10.010. 35949981 10.1016/j.aninu.2021.10.010 PMC9344316

[56] Grainger TN, Letten AD, Gilbert B, Fukami T. Applying modern coexistence theory to priority effects. Proc Natl Acad Sci U S A. 2019;116(13):6205–10. 10.1073/pnas.1803122116. 30850518 10.1073/pnas.1803122116 PMC6442631

[57] Sakamoto M, Ikeyama N, Ogata Y, Suda W, Iino T, Hattori M, et al. *Alistipes communis* sp. nov., *Alistipes dispar* sp. nov. and *Alistipes onderdonkii* subsp. *vulgaris* subsp. nov., isolated from human faeces, and creation of *Alistipes onderdonkii* subsp. *onderdonkii* subsp. nov. Int J Syst Evol Microbiol. 2020;70(1):473–80. 10.1099/ijsem.0.003778. 31633480 10.1099/ijsem.0.003778

[58] Buchenauer L, Haange S-B, Bauer M, Rolle-Kampczyk UE, Wagner M, Stucke J, et al. Maternal exposure of mice to glyphosate induces depression-and anxiety-like behavior in the offspring via alterations of the gut-brain axis. Sci Total Environ. 2023;905:167034. 10.1016/j.scitotenv.2023.167034. 37709081 10.1016/j.scitotenv.2023.167034

[59] Ruan D, Fan Q, Zhang S, Ei-Senousey H, Fouad A, Lin X, et al. Dietary isoleucine supplementation enhances growth performance, modulates the expression of genes related to amino acid transporters and protein metabolism, and gut microbiota in yellow-feathered chickens. Poult Sci. 2023;102(8):102774. 10.1016/j.psj.2023.102774. 37302324 10.1016/j.psj.2023.102774 PMC10276271

[60] Fung TS, Ryu KW, Thompson CB. Arginine: at the crossroads of nitrogen metabolism. EMBO J. 2025;44:1275–93. 10.1038/s44318-025-00379-3. 10.1038/s44318-025-00379-3 PMC11876448 39920310

[61] Liu G, Fang T, Yan T, Jia G, Zhao H, Huang Z, et al. Metabolomic strategy for the detection of metabolic effects of spermine supplementation in weaned rats. J Agric Food Chem. 2014;62(36):9035–42. 10.1021/jf500882t. 25162370 10.1021/jf500882t

[62] Seiler N, Knödgen B, Gittos MW, Chan W, Griesmann G, Rennert OM. On the formation of amino acids deriving from spermidine and spermine. Biochem J. 1981;200(1):123–32. 10.1042/bj2000123. 7332535 10.1042/bj2000123 PMC1163510

[63] Laudadio V, Passantino L, Perillo A, Lopresti G, Passantino A, Khan R, et al. Productive performance and histological features of intestinal mucosa of broiler chickens fed different dietary protein levels. Poult Sci. 2012;91(1):265–70. 10.3382/ps.2011-01675. 22184453 10.3382/ps.2011-01675

[64] De Verdal H, Mignon-Grasteau S, Jeulin C, Le Bihan-Duval E, Leconte M, Mallet S, et al. Digestive tract measurements and histological adaptation in broiler lines divergently selected for digestive efficiency. Poult Sci. 2010;89(9):1955–61. 10.3382/ps.2010-813. 20709981 10.3382/ps.2010-813

[65] Liao Y, Chen Q, Liu L, Huang H, Sun J, Bai X, et al. Amino acid is a major carbon source for hepatic lipogenesis. Cell Metab. 2024;36(11):2437-48. E8. 10.1016/j.cmet.2024.10.001. 39461344 10.1016/j.cmet.2024.10.001

[66] Handzlik MK, Gengatharan JM, Frizzi KE, McGregor GH, Martino C, Rahman G, et al. Insulin-regulated serine and lipid metabolism drive peripheral neuropathy. Nature. 2023;614:118–24. 10.1038/s41586-022-05637-6. 36697822 10.1038/s41586-022-05637-6 PMC9891999

