# Supplementary material for: Microbiota transplantation and multi-omics profiling integration unveil the mechanism of Alistipes communis-driven abdominal fat deposition in chickens
Source: J Anim Sci Biotechnol. 2026 May 20;17:98. doi: 10.1186/s40104-026-01439-2 (PMC13188826; doi:10.1186/s40104-026-01439-2)
Supplement: Supplementary file 2 — Additional file 2: Fig. S1 Comparative analysis of cecal microbial diversity and composition between donor and recipient broiler chickens in the cecal microbiota transplantation experiment. Fig. S2 Cecal microbial composition of experimental donor broiler chickens in the metagenome. Fig. S3 Comparative analysis of cecal microbiota composition in experimental donor broiler chickens from metatranscriptomic data. Fig. S4 The microbial diversity comparison after antibiotics treatments. Fig. S5 Differential gene expression analysis of cecal mucosa in recipient broiler chickens following Alistipes communis transplantation. [file 40104_2026_1439_MOESM2_ESM.docx]

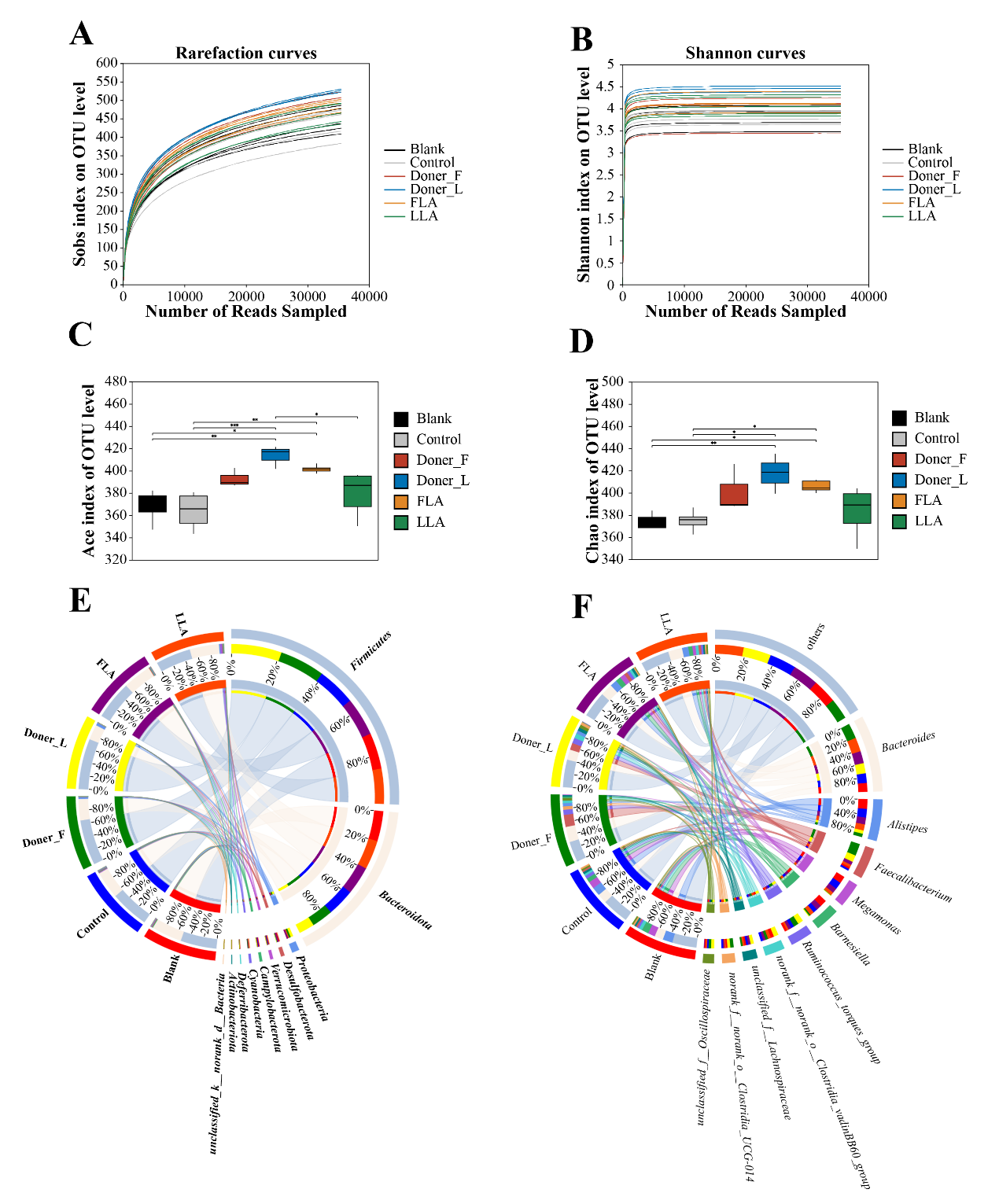


**Fig. S1** Comparative analysis of cecal microbial diversity and composition between donor and recipient broiler chickens in the cecal microbiota transplantation experiment. **A** and **B** The rarefaction analyses of species richness and Shannon diversity index. **C** and **D** Comparative analyses of the ACE and Chao diversity indices of cecal microbiota in donor and recipient chickens. **E** and **F** The major microbial composition at the phylum and genus levels in the cecal microbiota of donor and recipient chickens


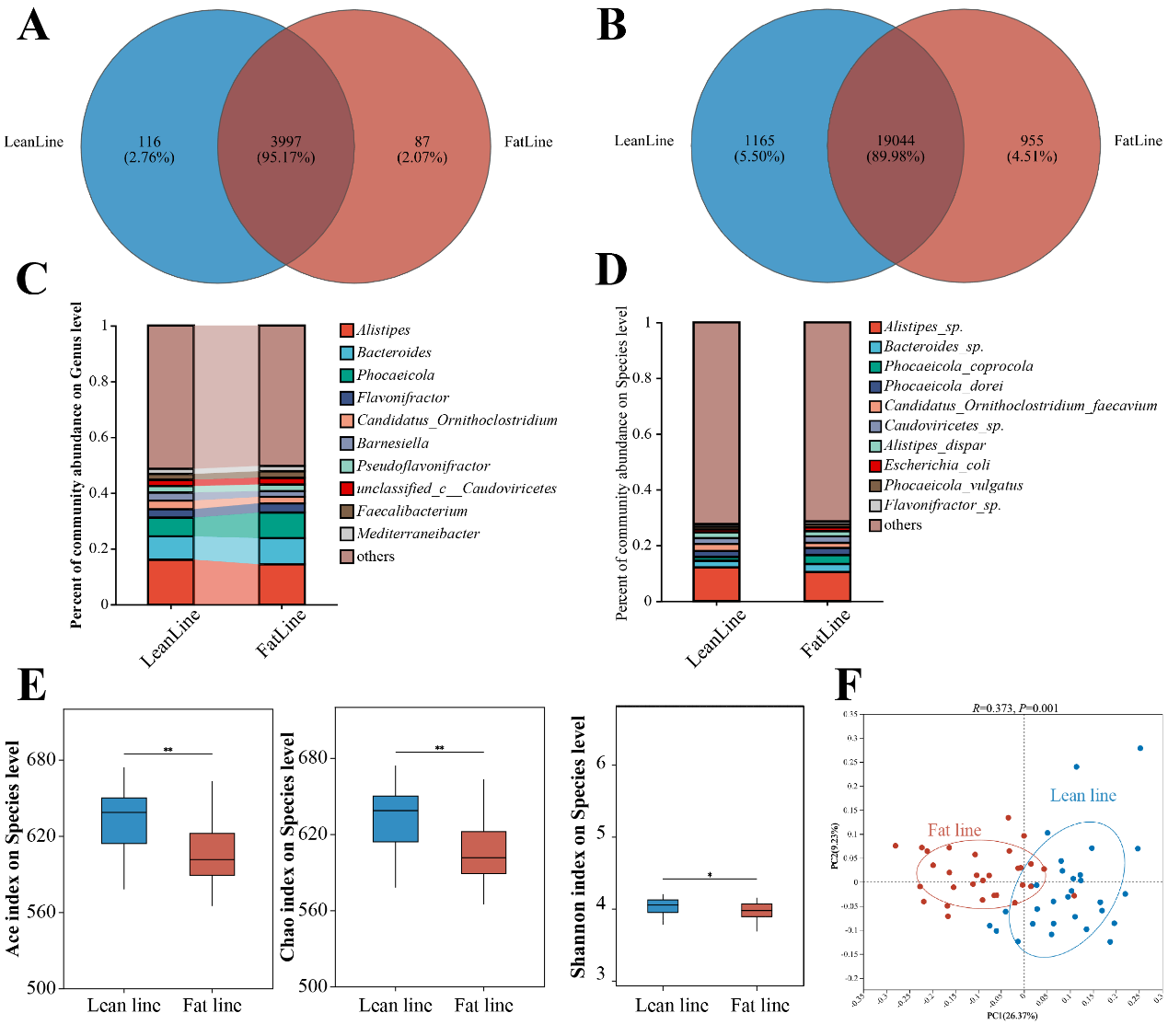


**Fig. S2** Cecal microbial composition of experimental donor broiler chickens in the metagenome. **A** and **B** Venn diagrams of cecal microbial genera and species, respectively, for the two broiler lines. **C** and **D** The major cecal microbial composition at the genus and species levels for the two broiler lines. **E** The comparative analysis of alpha diversity of cecal microbial species in donor broiler chickens from high- and low-fat lines. **F** PCoA analysis of cecal microbial species composition in donor broiler chickens from high- and low-fat lines


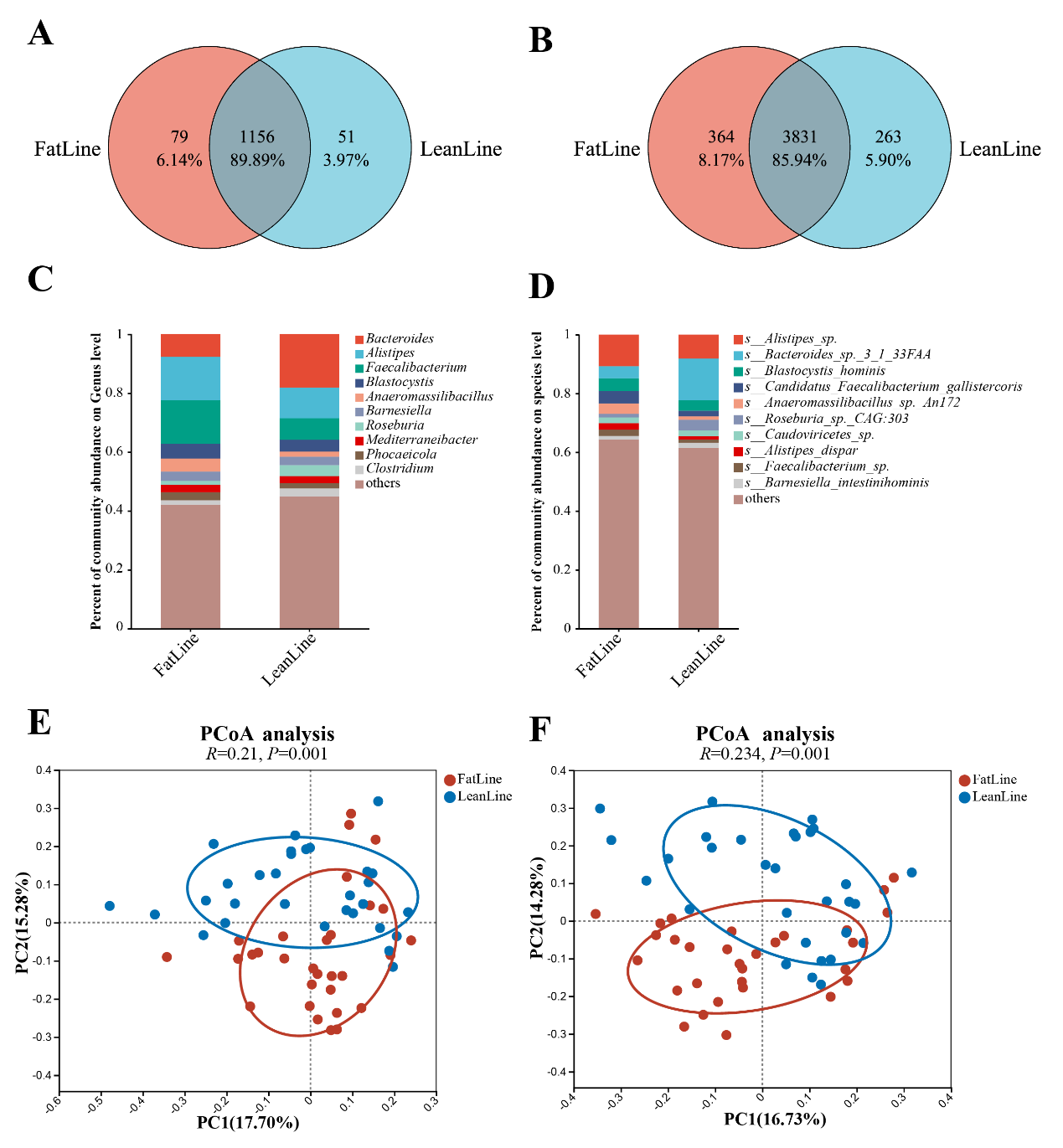


**Fig. S3** Comparative analysis of cecal microbiota composition in experimental donor broiler chickens from metatranscriptomic data. **A** and **B** Venn diagrams illustrating the cecal microbial genera and species classification, respectively, for experimental donor broiler chickens. **C** and **D** The composition of cecal microbiota at the genus and species levels in experimental donor broiler chickens. **E** and **F** PCoA analyses of cecal microbial composition at the genus and species levels between the two lines of experimental donor broiler chickens


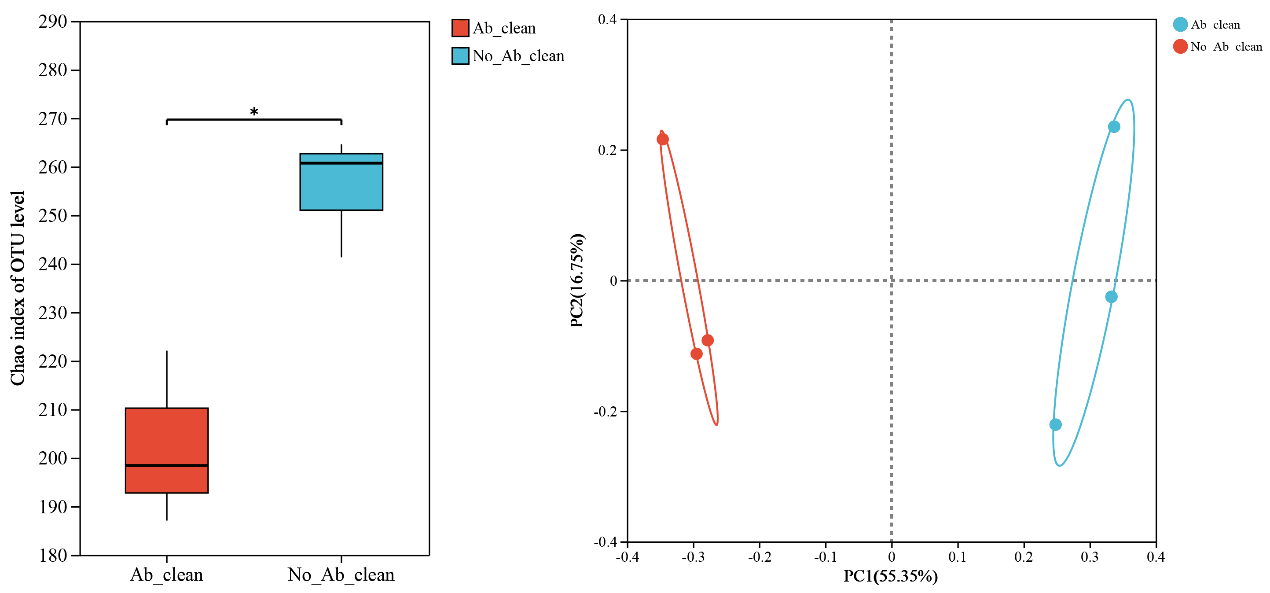


**Fig. S4** The microbial diversity comparison after antibiotics treatments. The left part was the observed numbers of OTUs, while the right part was the PCoA of OTUs composition between groups


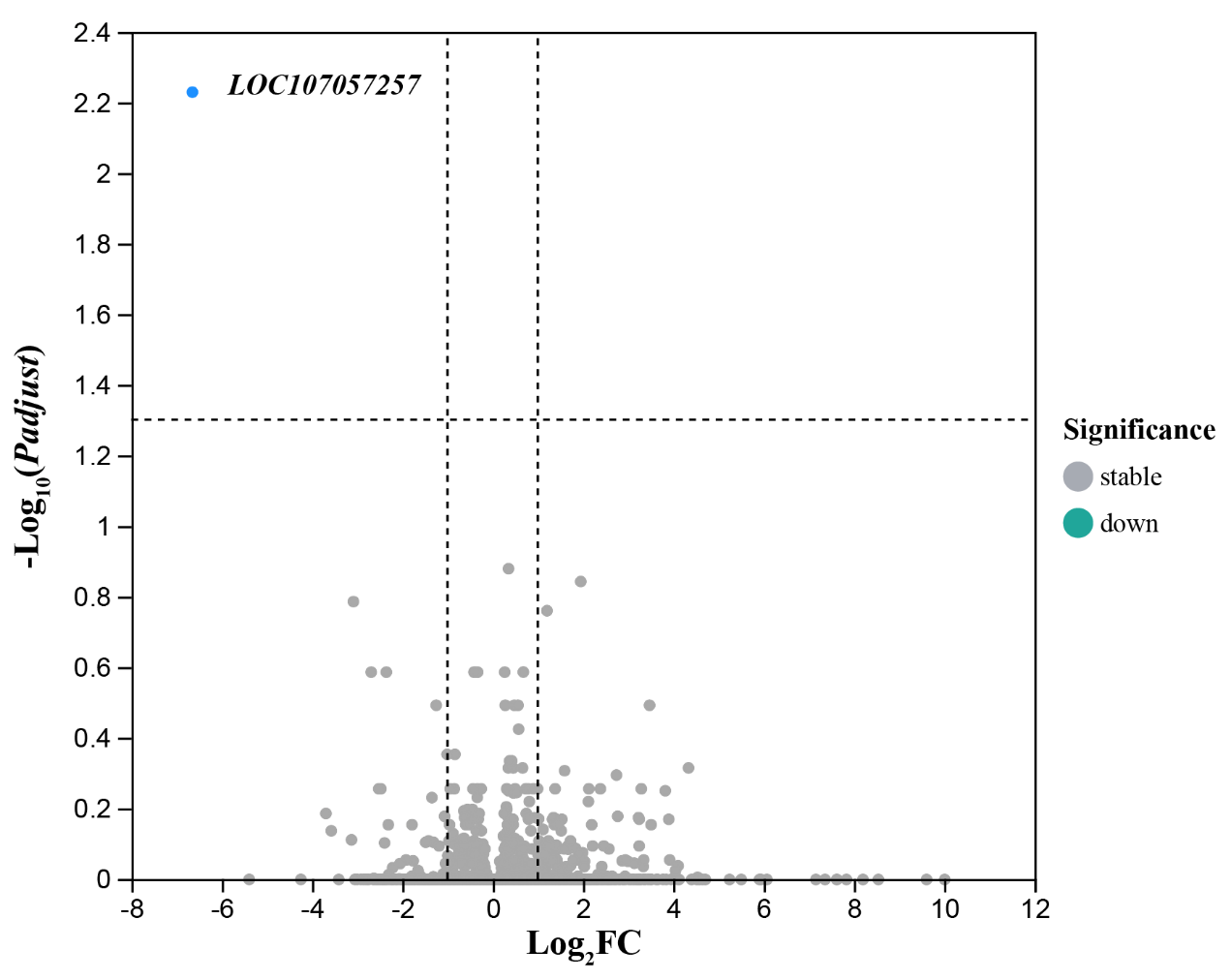


**Fig. S5** Differential gene expression analysis of cecal mucosa in recipient broiler chickens following *Alistipes communis* transplantation
